# Psychological traits associated with mental and performance resilience in long-distance and ultra-endurance runners: a systematic review

**DOI:** 10.3389/fpsyg.2026.1919146

**Published:** 2026-08-28

**Authors:** Qingyang Song, Yong-Gwan Song

**Affiliations:** 1 School of General Education, Huanghe Transportation University, Jiaozuo, Henan, China; 2 Pukyong National University, Busan, Republic of Korea

**Keywords:** athletic performance, resilience, running, sports psychology, ultramarathon

## Abstract

**Objective:**

Evidence on psychological traits in distance running remains fragmented across constructs, race contexts, and outcome definitions, while previous syntheses have generally addressed mental health, motivation, personality, or sport resilience separately. This review aimed to integrate and critically appraise psychological traits associated with both mental adaptation and performance continuity in long-distance and ultra-endurance runners while mapping concentrations and gaps in the evidence.

**Methods:**

This systematic review, reported in accordance with PRISMA 2020, searched PubMed, Scopus, and Web of Science on 10 June 2026. Eligible empirical studies examined distance-running populations and associations between psychological traits or trait-like constructs and mental or performance resilience outcomes. Risk of bias was assessed using Joanna Briggs Institute checklists. The evidence was synthesized narratively and mapped by construct and outcome domain.

**Results:**

The searches yielded 469 records; after 40 duplicates were removed, 429 records were screened, 89 full-text reports were assessed, and 53 studies were included. Most studies were observational, using cohort or cross-sectional designs; the remainder used qualitative or mixed-methods approaches. Maladaptive pain coping was most consistently associated with poorer pain adaptation and a lower likelihood of race completion. Emotional intelligence and emotion regulation were primarily associated with mood and stress outcomes, whereas motivation, personality, perfectionism, mental toughness, resilience, and self-efficacy showed heterogeneous associations. Critical appraisal identified minor concerns in three studies, moderate concerns in 36, and serious concerns in 14.

**Conclusion:**

By jointly mapping mental adaptation and performance continuity outcomes, this review clarifies recurring domain-specific associations and identifies major gaps in the endurance running psychology literature. However, 50 of the 53 included studies had moderate or serious methodological concerns, and the evidence was predominantly observational. The findings should therefore be interpreted as associations rather than causal effects and do not establish that modifying mental toughness or other psychological traits through training improves endurance running performance.

**Systematic review registration:**

https://osf.io/mxce4

## 1 Introduction

Long-distance and ultramarathon running are endurance disciplines in which performance depends on physiological capacity, training history, pacing, nutrition, and psychological regulation under sustained physical and cognitive demands (Berger et al., 2024; Knechtle and Nikolaidis, 2018). Ultra-endurance running is commonly defined as either a distance exceeding the marathon distance of 42.195 km or an event lasting approximately 6 h or longer (Knechtle, 2012; Scheer et al., 2022). In these contexts, resilience may help athletes respond adaptively to sport-related demands and adversity (Gupta and McCarthy, 2022; Sarkar and Fletcher, 2014). In sport psychology, resilience is conceptualized as adaptive functioning under stress, supported by interacting protective factors such as positive personality characteristics, motivation, confidence, focus, and social support (Gupta and McCarthy, 2022; Sarkar and Fletcher, 2014). Mental toughness is a related but distinct construct that has been studied as a psychological capacity relevant to coping with challenges, pressure, and sustained performance demands (Gucciardi, 2017; Gucciardi et al., 2017).

Accordingly, psychological adaptation in endurance running cannot be adequately represented by a single trait or performance endpoint. Relevant characteristics include mental toughness, self-efficacy, psychological resilience, emotional intelligence, motivation, passion, coping style, personality dimensions, pain-related beliefs, and cognitive control processes (Brace et al., 2020; Cona et al., 2015; Freund et al., 2013; Gameiro et al., 2023; Lane and Wilson, 2011; Partyka and Waśkiewicz, 2024). These characteristics have been examined in relation to mental adaptation outcomes, including mood, perceived stress, coping, recovery, and mental health symptoms, as well as performance continuity outcomes such as race completion, withdrawal, pacing, rank, finishing time, and performance satisfaction (Brace et al., 2020; Cona et al., 2015; Méndez-Alonso et al., 2021; Thuany et al., 2023). Distinguishing these domains is important because a psychological characteristic associated with more effective coping or better emotional regulation may not necessarily predict faster performance. Similarly, persistence cannot be assumed to be uniformly adaptive when continued effort may involve ignoring injury symptoms or other indicators of medical risk (Berger et al., 2024; Freund et al., 2013; Scheer et al., 2022).

The empirical literature reflects this complexity. Mental toughness, resilience, and self-efficacy have been associated with performance or completion in some running populations, but these relationships have not been consistently reproduced across events, athlete levels, or objective performance measures (Brace et al., 2020; Gameiro et al., 2023; Méndez-Alonso et al., 2021). Other studies have examined emotional intelligence, pain tolerance, coping, motivation, personality, and cognitive functioning, further demonstrating that the psychological demands of endurance running extend beyond mental toughness alone (Cona et al., 2015; Freund et al., 2013; Lane and Wilson, 2011). This variability in constructs, populations, and outcomes makes individual findings difficult to interpret in isolation and creates a need for a synthesis that retains their domain-specific and methodological context.

At the review level, related evidence has also remained compartmentalized. Previous syntheses have addressed mental health in ultra-endurance runners, motivation in marathon and ultramarathon runners, personality among marathon runners, and resilience across sport performers (Braschler et al., 2024; Gupta and McCarthy, 2022; Partyka and Waśkiewicz, 2024; Thuany et al., 2023). These reviews have advanced understanding within their respective domains, but they have not jointly examined how a broad range of trait-level psychological characteristics relate to both mental adaptation and performance continuity specifically in long-distance and ultra-endurance running.

The novelty of the present review lies in integrating these two previously separated strands of evidence within a single framework. In addition to synthesizing associations across long-distance, trail, ultramarathon, and ultra-endurance populations, the review distinguishes between mental resilience outcomes and performance resilience outcomes, incorporates qualitative evidence concerning runners’ experiences and interpretations, critically appraises studies using design-appropriate Joanna Briggs Institute checklists, and maps the distribution of evidence across psychological constructs and outcome domains. Its contribution is therefore not to establish that particular traits cause superior performance, but to clarify where associations recur, where findings remain inconsistent, and which construct–outcome relationships require more rigorous prospective or intervention-based investigation. Accordingly, this review aimed to identify, critically appraise, and synthesize empirical studies evaluating psychological traits and trait-like attributes associated with mental and performance resilience in long-distance and ultra-endurance runners.

## 2 Methods

### 2.1 Design

This systematic review was conducted and reported in accordance with the PRISMA 2020 statement, and the search reporting followed principles from the PRISMA-S extension (Page et al., 2021; Rethlefsen et al., 2021). The review examined empirical evidence on psychological traits and trait-like attributes associated with mental and performance resilience among long-distance and ultra-endurance runners. The protocol was registered prospectively on the Open Science Framework before the database searches were conducted (osf.io/mxce4; June 10, 2026).

### 2.2 Eligibility criteria

Eligible studies were original empirical investigations involving human participants described as long-distance, endurance, marathon, trail, ultramarathon, ultrarunning, or ultra-endurance runners. For this review, long-distance running encompassed samples explicitly described by the study authors as distance, endurance, marathon, trail, or long-distance runners; ultra-endurance running encompassed races longer than 42.195 km or events described as lasting at least 6 h. Studies of mixed endurance sport samples were eligible only when runner-specific data were reported separately or could be extracted without inference. Studies primarily examining non-running endurance disciplines, recreational physical activity without a distance-running classification, or clinical exercise programs that did not involve distance-running populations were excluded.

Eligible exposures were psychological traits or trait-like constructs assessed before, during, or after participation in running. These included, but were not limited to, mental toughness, resilience, self-efficacy, self-regulation, motivation, passion, grit, perseverance, hardiness, personality dimensions, emotional intelligence, coping style, pain tolerance, pain-related anxiety, mindfulness, optimism, confidence, and cognitive control characteristics when conceptualized as relatively stable individual differences or predictors of adaptation. Eligible mental resilience outcomes included psychological resilience scores, mental health symptoms, wellbeing, stress tolerance, affective stability, mood recovery, coping under adversity, pain coping, and recovery-related psychological outcomes. Eligible performance resilience outcomes included race completion or non-completion, withdrawal, rank, race time, pacing maintenance, performance satisfaction, maintenance of performance under fatigue or adverse conditions, and other explicitly defined outcomes reflecting performance continuity.

Eligible designs included quantitative observational studies, intervention studies reporting baseline or follow-up associations relevant to the review question, mixed-methods studies, and qualitative studies that directly addressed trait-related resilience in running contexts. Quantitative studies were required to report an association, comparison, prediction model, effect estimate, or sufficient data to characterize the relationship between at least one psychological trait and at least one eligible mental or performance outcome. Qualitative studies were synthesized separately unless they contained extractable quantitative data. Reviews, editorials, commentaries, letters without original data, conference abstracts lacking sufficient methodological information, case reports, and non-peer-reviewed material were excluded. No restrictions were imposed on publication date or language.

### 2.3 Information sources

PubMed, Scopus, and Web of Science were searched on June 10, 2026. The reference lists of included studies and closely related reviews were also screened.

### 2.4 Search strategy

The search strategy comprised three concept blocks: the running population, psychological traits or resilience-related constructs, and mental health or performance-related outcomes. The strategy was iteratively refined after reviewing terminology related to definitions and performance limits in ultra-endurance running; mental toughness and self-efficacy in ultramarathon runners; mental toughness and resilience in trail runners; psychological predictors in ultra-trail events; trait emotional intelligence; pain tolerance and personality; cognitive functioning; motivation; and mental health in ultra-endurance runners.

([Title/abstract or Topic] marathon OR ultramarathon OR ultra-marathon OR ultrarunner OR ultrarunning OR ultra-endurance OR ultraendurance OR “ultra endurance” OR “endurance running” OR “endurance run” OR “long-distance running” OR “long-distance run” OR “long distance running” OR “long distance run” OR “distance run” OR “distance running” OR “trail run” OR “trail running” OR “trail runner”)

AND

([Title/abstract or Topic] personality OR resilience OR resilient OR “self efficacy” OR “self-efficacy” OR “emotional intelligence” OR motivation OR motivational OR grit OR mindfulness OR passion OR perseverance OR persistence OR “self-regulation” OR “self regulation” OR “goal orientation” OR “pain tolerance” OR coping OR hardiness OR optimism OR “cognitive control” OR “executive function” OR “mental toughness”)

AND

([Title/abstract or Topic] performance OR “race performance” OR “race time” OR “finish time” OR completion OR rank OR ranking OR finish OR “did not finish” OR dropout OR adaptation OR recovery OR fatigue OR wellbeing OR stress OR anxiety OR mood OR “mental health” OR injury)

Search results were exported with full bibliographic data, abstracts, keywords, and database identifiers where available.

### 2.5 Selection process

Records retrieved from all databases were imported into EndNote and deduplicated using automated and manual procedures. Two authors independently screened titles and abstracts against the eligibility criteria using a standardized Excel form. Each author recorded an initial decision without access to the other author’s assessment. After completing the screening for titles and abstracts, the decisions were compared, and discordant records were discussed individually. For each disagreement, the authors jointly re-examined the title and abstract and applied the prespecified population, exposure, outcome, and design criteria. When the available information was insufficient to justify exclusion, the report was retained for full-text assessment. The same authors independently assessed the full texts of potentially eligible studies before comparing their eligibility decisions. Full-text disagreements were resolved by jointly re-examining the complete report, identifying the specific eligibility criterion responsible for the disagreement, and reaching a consensus decision based on the prespecified criteria. No third author was involved, and no disagreements remained unresolved after this process. Reasons for exclusion at the full-text stage were recorded and summarized in the PRISMA flow diagram.

### 2.6 Data extraction and data collection process

A standardized, pilot-tested extraction form was used. Two authors independently extracted data from each included study, with the initial extraction forms completed separately before comparison. Discrepancies were resolved by consensus through re-examination of the source report and the operational definitions in the extraction form. The form was piloted on a small sample of included studies and refined before full data extraction.

When information considered necessary for determining eligibility, characterizing the study, interpreting an eligible exposure–outcome association, or completing the methodological appraisal was missing or unclear, the corresponding author was contacted using the most recent email address available. An initial request was followed by one reminder after seven days if no response had been received. This time was provided to ensure the authors were fully aware of the request. Any information supplied by study authors was checked against the published report and incorporated into the extraction form with its source documented. When no response was received within this period, the study was retained and synthesized using the information available in the published report. Missing information was recorded as not reported or unclear, and no values were imputed. Corresponding authors were contacted for two included studies. Responses were received for both studies.

### 2.7 Data items

Extracted items included bibliographic details, country, study design, recruitment source, context, participant characteristics, sample size, age, sex distribution, competitive level, running distance or event type, surface or terrain, race format, prior running experience, training volume, inclusion and exclusion criteria, psychological constructs, measurement instruments, assessment timing, mental resilience outcomes, performance resilience outcomes, statistical methods, covariates, reported effect estimates, uncertainty intervals, *p*-values where provided, and the authors’ conclusions. For longitudinal or intervention studies, baseline characteristics, follow-up intervals, exposure timing, attrition, and intervention characteristics were also extracted. For qualitative studies and the qualitative components of mixed-methods studies, extracted information additionally included the stated qualitative methodology, data-collection approach, analytic method, reported themes or categories, and the authors’ principal interpretive findings.

### 2.8 Outcomes

The primary outcome domains were mental resilience and performance resilience. Mental resilience encompassed validated resilience scores, psychological adaptation to stressors, mental health symptoms, wellbeing, affective responses, mood recovery, perceived stress, coping effectiveness, pain coping, and related outcomes explicitly linked to adaptation or psychological functioning. Performance resilience encompassed race completion, withdrawal or non-completion, pacing maintenance, adaptive goal adjustment, performance satisfaction under demanding conditions, and the preservation of performance during fatigue, pain, sleep deprivation, environmental exposure, injury risk, or other race-related stressors. An outcome was classified as a direct indicator of performance resilience when it captured persistence, maintenance, adaptation, or successful task continuation in relation to an identifiable stressor or performance disruption. Race time, finishing position, rank, or average speed reported without an explicit adversity, deterioration, continuity, or adaptation component was classified as general performance outcomes. These measures were retained because they were relevant to the broader relationship between psychological characteristics and running performance, but they were interpreted as performance correlates rather than direct indicators of performance resilience.

### 2.9 Risk of bias and methodological quality assessment

Risk of bias and methodological limitations were assessed independently by two authors using design-appropriate Joanna Briggs Institute critical appraisal checklists. The authors completed their initial item-level judgments separately and without access to one another’s ratings. Analytical cross-sectional studies were assessed using the JBI Critical Appraisal Checklist for Analytical Cross-Sectional Studies (Munn et al., 2023), while cohort and longitudinal studies were assessed using the JBI Critical Appraisal Checklist for Cohort Studies (Barker et al., 2025). Each checklist item was recorded as “Yes,” “No,” “Unclear,” or “Not applicable,” together with a brief rationale supporting the judgment. Disagreements were resolved through discussion and consensus, with the published report and the review-defined appraisal criteria re-examined before a final judgment was assigned. No external adjudicator was involved.

No numerical methodological quality score or percentage cut-off was calculated. Overall study-level judgments were review-defined and based on the nature, severity, and likely consequence of the identified limitations for the eligible exposure–outcome association or qualitative interpretation. Minor methodological concerns were assigned when the key validity domains were adequately addressed and any remaining limitations were unlikely to alter the findings. Moderate methodological concerns were assigned when one important limitation or several lesser limitations reduced confidence in the findings but did not seriously undermine the principal interpretation. Serious methodological concerns were assigned when a major limitation in a core domain or multiple interacting limitations were considered likely to distort the reported association or interpretation.

For quantitative studies, the principal domains considered were participant selection and temporal ordering, validity and reliability of exposure measurement, validity and reliability of outcome measurement, identification and management of confounding, follow-up and missing data, and appropriateness of the statistical analysis. For qualitative studies, the principal considerations were congruity among the research question, methodology, data collection, analysis, and interpretation; representation of participants’ voices; researcher positioning and reflexivity; ethical reporting; and whether the conclusions were supported by the analysis. When more than one checklist was applied to an article, the article-level judgment reflected the most consequential limitations across its eligible components rather than an average of checklist responses. Overall judgments were not used as automatic exclusion criteria; instead, the item-level and overall appraisal findings informed the interpretation of and confidence in the evidence.

Inter-author agreement was evaluated using the original decisions before consensus. For title/abstract and full-text eligibility decisions, observed Cohen’s kappa was *κ* = 0.93. At full-text assessment, agreement was κ = 0.95. Agreement estimates were calculated from the original independent decisions before consensus.

### 2.10 Effect measures

For quantitative studies, extracted effect measures included correlation coefficients, standardized beta coefficients, unstandardized regression coefficients, odds ratios, risk ratios, mean differences, standardized mean differences, and hazard ratios, as applicable. When sufficient information was available, correlations were transformed to Fisher’s z values for quantitative synthesis and back-transformed for interpretation. For dichotomous outcomes such as completion versus non-completion, odds ratios or risk ratios were extracted or calculated when possible. When both adjusted and unadjusted estimates were reported, both were extracted; adjusted estimates were prioritized in the primary synthesis when the covariate adjustment was conceptually appropriate and comparable across studies.

### 2.11 Synthesis methods

The primary synthesis was narrative and structured by psychological construct, population or race type, outcome domain, and study design. Tables summarized study characteristics, instruments, outcomes, effect estimates, and risk-of-bias judgments. A structured narrative synthesis described the direction, magnitude, precision, and consistency of the reported associations. Potential sources of heterogeneity were explored narratively. Given the predominance of observational designs and the heterogeneity of the psychological constructs and outcomes, the synthesis was intended to characterize associations and evidence gaps. It was not designed to estimate causal effects of psychological traits or the effects of interventions intended to modify those traits.

A secondary risk-of-bias sensitivity synthesis was conducted after excluding studies classified as having serious methodological concerns. Each principal synthesis finding was reassessed using only studies with minor or moderate methodological concerns. For each finding, we compared the number and design of contributing studies, the direction and consistency of associations, reported effect estimates and uncertainty where available, and the extent to which the original interpretation remained supported. Findings were classified as retained when their direction and interpretation remained supported, attenuated when the supporting evidence became substantially narrower or less consistent, and not robust when all or nearly all supporting evidence came from studies with serious methodological concerns. Studies with serious concerns remained in the primary evidence tables but did not contribute to the restricted sensitivity synthesis.

### 2.12 Certainty of the evidence

The certainty of the evidence supporting the principal quantitative findings was assessed using the Grading of Recommendations Assessment, Development and Evaluation (GRADE) approach (Brignardello-Petersen and Guyatt, 2025). Certainty was evaluated at the level of each defined body of evidence rather than for individual studies or for the review as a whole. The evidence bodies were specified before the certainty ratings were assigned and represented the principal quantitative associations underpinning the review findings and conclusions, i.e., maladaptive pain coping with pain interference; maladaptive pain coping with race completion; emotional intelligence or emotion regulation with mood- and stress-related outcomes; motivational quality or irrational performance beliefs with psychological wellbeing, anxiety, or perceived health; mental toughness, resilience, or self-efficacy with completion or performance outcomes; perfectionistic characteristics with general running performance; and psychological characteristics with injury or risk-related continuation.

The target of each certainty assessment was to determine confidence in the presence and direction of an association between a psychological characteristic and an eligible outcome. It was not about confidence in a causal effect or in the effectiveness of an intervention intended to modify that characteristic. Because the studies reported heterogeneous effect measures and no common clinically meaningful effect threshold was available, the null association was used as the threshold of interest. The assessments, therefore, addressed whether the true association was likely to lie in the reported direction rather than whether it exceeded a common magnitude across studies. Consequently, a higher certainty rating for an association would not establish that the psychological characteristic caused the outcome or that changing the characteristic through training would improve performance or mental health.

Following the target-of-certainty approach, observational evidence was considered an appropriate starting source for evaluating the defined associations. Each evidence body initially entered the assessment at high certainty when the contributing study designs were appropriate to the associational question and was subsequently rated down when concerns were identified. Certainty could be reduced by one or two levels for serious or very serious concerns, respectively, across five domains: risk of bias, inconsistency, indirectness, imprecision, and publication bias. Final certainty was categorized as high, moderate, low, or very low.

Risk of bias was assessed using item-level judgments from the design-appropriate Joanna Briggs Institute critical appraisal checklists. The review-defined overall categories of minor, moderate, or serious methodological concerns were considered alongside the specific limitations affecting each exposure–outcome association; they were not used as automatic numerical rules for downgrading. Particular attention was given to participant selection, the validity and reliability of psychological and outcome measurements, temporal ordering, management of confounding, completeness of follow-up, missing data, and the appropriateness of the statistical analyses.

Inconsistency was evaluated by comparing the direction, magnitude, and statistical compatibility of associations across the studies contributing to each evidence body. Differences in the running population, competitive level, event format, psychological instrument, outcome definition, assessment timing, and covariate adjustment were considered when determining whether variation could be explained. When only one study contributed to an evidence body, inconsistency could not be evaluated directly; the absence of replication was documented but was not, by itself, treated as evidence of inconsistency.

Indirectness was assessed against the population, psychological exposure, outcome, and associational target specified for each finding. Ratings considered whether the available samples represented the long-distance and ultra-endurance running populations of interest, whether different instruments measured sufficiently comparable psychological constructs, and whether the outcomes directly represented mental or performance resilience. Generic performance outcomes (including race time, rank, placing, or speed without an explicit continuity, adaptation, or adversity component) were considered indirect measures of performance resilience.

Imprecision was evaluated using the number of contributing studies and participants, the number of outcome events where applicable, reported confidence intervals, and the extent to which the available results were compatible with materially different interpretations. Where confidence intervals were unavailable, imprecision was judged based on the sample size, distribution of outcomes, reported effect estimates, and statistical information presented by the primary studies. Certainty was reduced when the available evidence was too sparse or inadequately reported to distinguish a meaningful association from a null or substantially different association.

Publication bias was considered qualitatively because the number of studies within each evidence body was insufficient. Judgments considered the predominance of small or single-event studies, concentration of evidence within closely related research groups or settings, selective reporting of outcomes or analyses, and the possibility that null or unfavorable associations remained unpublished. No rating was increased based on large effects, dose–response gradients, or residual confounding unless these features were clearly demonstrated and could not be explained by methodological limitations.

Studies were grouped according to psychological exposure, outcome domain, and conceptual comparability. For each evidence body, the reviewers considered all available estimates, including their direction, magnitude, uncertainty, methodological context, and consistency, rather than relying on statistical significance or vote counting. A risk-of-bias sensitivity synthesis was also used to inform the certainty judgments. Each principal finding was reassessed after excluding the 14 studies classified as having serious methodological concerns. Stability after exclusion was considered when evaluating the risk-of-bias domain and the overall robustness of the finding but did not automatically prevent downgrading, because most studies remaining in the restricted synthesis still had moderate methodological concerns.

Two authors independently assessed each evidence body using a standardized GRADE evidence profile. They recorded the judgment for every domain, the number of levels downgraded, and an explicit rationale. The authors subsequently compared their assessments and resolved disagreements through discussion. The final ratings were presented in a summary-of-findings table together with explanatory footnotes supporting each downgrading decision.

GRADE terminology was used when interpreting the findings. High-certainty evidence was described as showing an association; moderate-certainty evidence as probably showing an association; low-certainty evidence as suggesting that an exposure may be associated with an outcome; and very-low-certainty evidence as indicating that the association was very uncertain. These expressions refer only to confidence in the defined association and do not imply causality.

## 3 Results

### 3.1 Study selection

**Figure 1:**
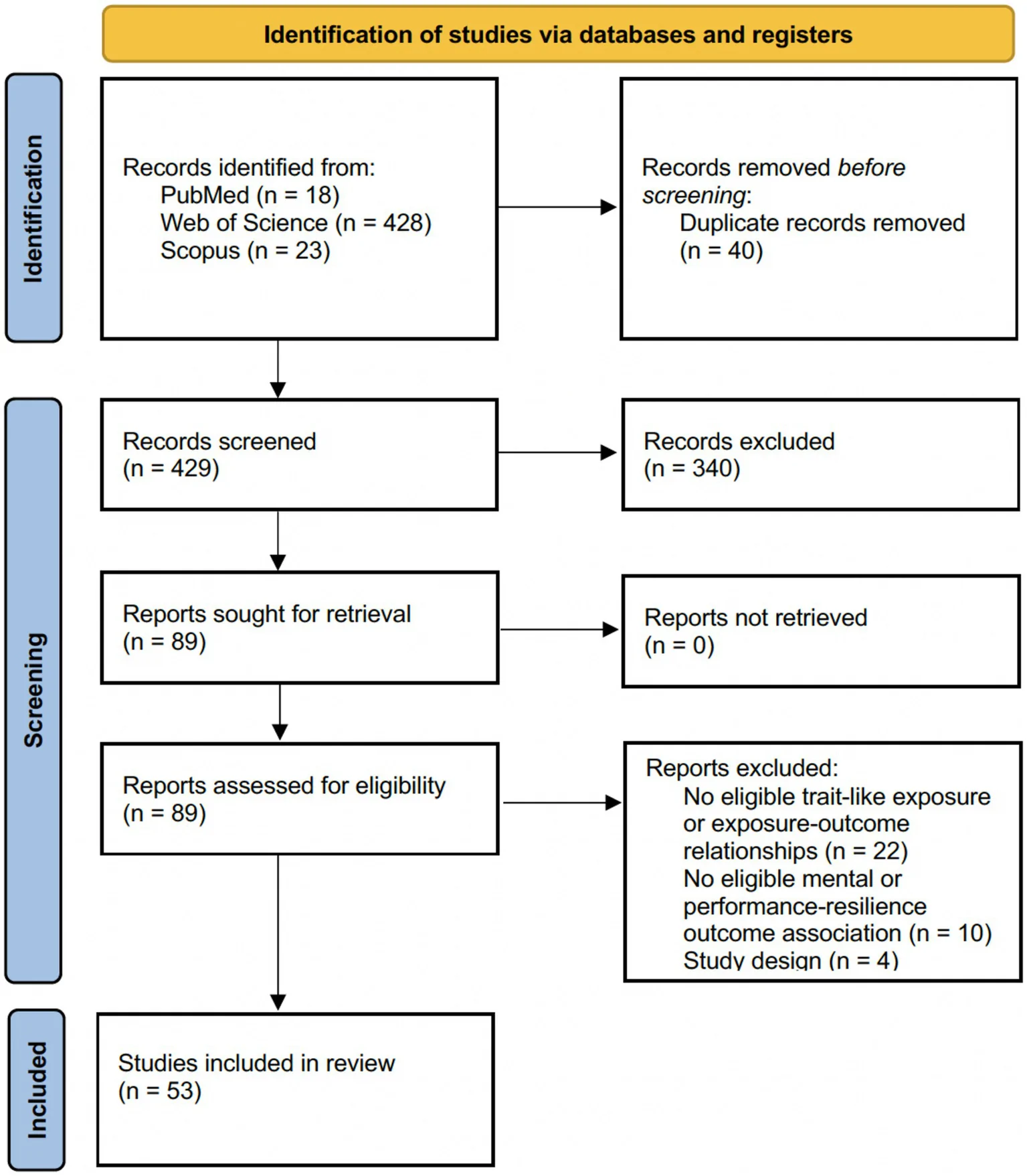
Study selection flow diagram.

The database searches yielded 469 records: 18 from PubMed, 428 from Web of Science, and 23 from Scopus. After 40 duplicates were removed, 429 unique records underwent title and abstract screening. Of these, 340 were excluded, leaving 89 reports for full-text eligibility assessment. At the full-text stage, 36 reports were excluded. The most common reason was the absence of an eligible psychological trait-like exposure or exposure–outcome relationship (22 reports), followed by the absence of an eligible association with a mental or performance resilience outcome (10 reports) and an ineligible publication type or study design (4 reports). The final synthesis included 53 studies. The selection process is summarized in Figure 1.

### 3.2 Characteristics of included studies

Table 1 summarizes the characteristics of the included studies. The studies were published between 1986 and 2026. Most used observational designs, including prospective or repeated-measures cohorts and analytical cross-sectional studies; the remainder used qualitative or mixed-methods designs.

**Table 1:** Characteristics of the included studies.

Study	Context	Population and sample	Study design	Main eligible outcome domain or domains
Alschuler et al. (2019)	Chile, China, and Namibia; 2016 RacingThePlanet self-supported 250 km, 6-stage desert ultramarathon events	204 adult ultramarathon entrants; mean age approximately 41 years; about 73% male	Prospective observational repeated-measures cohort	Percent time thinking about pain and pain interference
Alschuler et al. (2020)	Chile, China, and Namibia; 2016 RacingThePlanet self-supported 250 km, 6-stage desert ultramarathon events	204 adult ultramarathon entrants; mean age approximately 41 years; approximately 73% men; 176 finishers and 28 non-finishers	Prospective observational repeated-measures cohort	Race completion and finishing-position quintile
Beattie et al. (2025)	Portugal, Madeira Island Ultra Trail 115 km mountain ultramarathon; online surveys via race organizer	47 mountain ultramarathon runners with complete post-race questionnaires; mean age 42.85 years; 89.6% male	Mixed-methods observational study with a primarily quantitative component	Performance satisfaction, coping effectiveness, coping frequency trajectory, and qualitative coping/satisfaction themes
Bennett et al. (2026)	Privately sponsored six-day loop ultramarathon around a lake with extensive medical, crew, mental performance, sport-science, nutrition, and gear support	Seven self-identifying cisgender women ultrarunners; three elite and four non-elite; age range 27 to 48 years	Qualitative narrative constructionist study	Pain meaning-making, problem-focused coping, chosen pain, collective care, and sustainable engagement
Boere et al. (2026)	British Columbia, Canada; six 50-km ultramarathon races; testing tent near race start and finish	76 recreational runners completing the study; 39 women and 37 men; age approximately 37 years	Prospective observational pre- and post-race field study	Behavioral reaction time, reaction-time variability, accuracy, N2 amplitude and latency, P3 amplitude and latency
Brace et al. (2020)	United States, Hawaii; 2019 HURT100 100-mile mountain ultramarathon	56 elite ultramarathon runners; 38 men and 18 women; mean age 38.86 years	Cross-sectional observational study with pre-race online survey and official race outcomes	Ultra-Trail World Tour rank, HURT100 completion, finishing place, and finishing time
Brick et al. (2015)	Elite endurance runners recruited via national endurance coach and interviews connected to University of Limerick context	Elite endurance runners who had competed internationally and still competed in events from 3,000 m to ultra-distance; 6 women and 4 men; mean age 35.6 years	Qualitative semi-structured interview study with content analysis	Cognitive-strategy planning, monitoring, active self-regulation, distraction, review/evaluation, and metacognitive feelings and judgments
Buman et al. (2008)	United States; online marathon running listserves and web-based data collection	Experienced recreational marathon runners; 40 men and 17 women; mean age 41.79 years; all had prior hitting-the-wall experience	Qualitative web-based open-ended interview/survey using grounded theory procedures	Characteristics of hitting the wall and coping responses
Chalabaev et al. (2017)	France; Marathon des Alpes-Maritimes in Nice, with questionnaires completed the day before the race	Marathon runners; Study 1 *n* = 378, Study 2 *n* = 339; mean age approximately 43 years in both samples	Two cross-sectional correlational questionnaire studies	Perceived susceptibility to marathon-related injury and perceived likelihood of continuing to run through pain
Christensen et al. (2018)	United States; 2014 Western States Endurance Run	161 km ultramarathon runners; 189 questionnaire respondents and 152 finishers reported for finishing-time analysis	Observational survey study with multiple regression	161 km ultramarathon finishing time
Corrion et al. (2018)	France; Côte d’Azur Mercantour Ultra-Trail, 140 km with 10,000 m positive elevation	221 ultra-trail runners aged 18 to 61 years; 195 men and 26 women; 96 finishers and 125 non-finishers	Prospective observational questionnaire study	Race completion or withdrawal
Deaner et al. (2019)	Online international sample; mostly United States, United Kingdom, and Canada	Marathon runners aged at least 21 years; 1,342 with pacing data and 1,479 with all psychological constructs	Retrospective online survey with regression analyses	Second-half marathon slowing as pacing maintenance outcome
Diotaiuti et al. (2021)	Italy; Lazio Region running associations affiliated with the Italian Athletics Federation	750 endurance runners; mean age 42.58 years; 86.9% male; mixed running specialties	Cross-sectional questionnaire study with structural equation modeling	Homeostatic reintegration and resilient reintegration
Freund et al. (2013)	Germany/Switzerland participants; TransEurope FootRace 2009 context and University Hospital Ulm testing	11 male TransEurope FootRace ultramarathon runners and 11 matched healthy non-marathon controls	Matched case–control study with experimental cold pressor testing	Cold pain tolerance, Trait and Character Inventory personality traits, and general self-efficacy
Gameiro et al. (2023)	Portugal; Portuguese Trail Running Association and online recruitment	307 Portuguese trail runners with valid International Trail Running Association profile; mean age 41.98 years; 247 men and 60 women	Cross-sectional questionnaire study with structural equation modeling	Resilience score and International Trail Running Association Performance Index
Gauld et al. (2024)	France; follow-up after serious ultramarathon complications requiring intensive care or dialysis; race locations not fully reported	12 ultrarunners with serious post-ultramarathon complications; 11 men and 1 woman; mean age 43 years	Retrospective descriptive and network analysis	Exercise addiction classification, EAI item centrality, Big Five personality profile
Gayton et al. (1986)	United States; Casco Bay Marathon, Portland, Maine	33 marathon finishers; 22 men/11 women; mean age 38.6 years	Prospective observational correlational study	Predicted and actual marathon finishing times
Gillet et al. (2012)	Morocco; Marathon des Sables, Sahara Desert; participant countries not reported	151 long-distance runners after deletion of two outliers; Study 2 only	Prospective observational cluster-analytic study	Final ranking and emotional/physical exhaustion
Graham et al. (2021)	Canada; 6,633 Arctic Ultra-Marathon, Yukon and Northwest Territories; 120-mile stage in Arctic cold conditions	12 experienced ultramarathon runners; 9 men and 3 women; mean age 42 years	Prospective observational field cohort study	Injury rate, sleep duration, Brunel Mood Scale mood states, mental toughness correlations
Gross (2025)	Estonia; recreational ultrarunning community interviews, blogs, and participant observation	40 recreational ultrarunners/interviewees; 10 women and 30 men; age 26-55; plus approximately 20 running blogs and participant observation	Qualitative ethnographic study with phenomenological perspective and reflexive thematic analysis	Moral language, discourse about pain and suffering, and self-talk during pain and suffering
Howe et al. (2019)	United Kingdom; laboratory 80.5 km treadmill ultramarathon	12 ultramarathon runners analyzed; 9 men and 3 women; mean age 34 years; 2 of 14 initially recruited excluded for non-completion	Prospective observational laboratory time-trial study	Total mood disturbance, serum cortisol, perceived exertion, and completion time
Jackman et al. (2024)	Online and telephone event-focused interviews after recent excellent distance-running performances	21 long-distance runners with recent excellent performances; 6 women and 15 men; mean age 34.90 years; plus 10 external member-reflection participants	Qualitative event-focused interview study with time-ordered matrix analysis	Goal-striving decisions, goal revision, and action-crisis management
Jaenes et al. (2022)	Spain; Seville Marathon Expo before the 26th International Seville Marathon	238 adult male marathon runners aged 18-65 years; mean age 41.22 years; 86% had completed at least one previous marathon	Cross-sectional observational pre-race questionnaire study with moderated mediation analysis	Sport Anxiety Scale-2 somatic anxiety, worry, concentration disruption, and modeled overall competitive anxiety
Jowett et al. (2018)	United Kingdom; pre-marathon exhibition and online recruitment	224 marathon runners; 143 men and 81 women; mean age 39.77 years; prior marathoners and first-marathon preparers	Cross-sectional observational survey testing the 2 × 2 model of perfectionism	Brief COPE problem-focused, emotion-focused, and avoidance coping with injury
Kelemen et al. (2025)	Hungarian national/international-level distance running context.	16 Hungarian international-level distance runners; professional/semiprofessional university athletes served as controls.	Cross-sectional analytical/profile study.	Competitive anxiety domains (cognitive and somatic anxiety) and self-confidence. Restricted extraction; detailed study-level estimates were not available in the master extraction workbook.
Kilduff (2014)	United States; local running-club race results in a midsized town	Regular amateur runners in mixed-distance road races, 3.0 to 21.1 km; 82 regular runners in archival analysis	Retrospective archival observational repeated race-results study with runner pilot survey	Running pace in seconds per kilometer
Krokosz et al. (2018)	Poland; 100 km track ultramarathon in a sports stadium	20 experienced male marathon and ultramarathon runners aged 31 to 50 years	Double-blind randomized placebo-controlled vitamin D3 field study with psychological correlational analyses	UWIST Mood Adjective Check List energetic arousal, tense arousal, and hedonic tone
Lane and Wilson (2011)	United Kingdom; Marathon of Britain six-stage ultra-endurance foot race	34 ultra-endurance runners aged 23 to 59 years; reported sex counts were 24 men and 8 women but do not sum to 34	Prospective repeated-measures observational field study	Brunel Mood Scale anger, confusion, depression, fatigue, tension, and vigor; UWIST calmness and happiness items
Larumbe-Zabala et al. (2020)	Mexico; marathon training group and performance testing context, target marathon location not reported	16 healthy recreational marathon runners, 8 men and 8 women, preparing for the same major marathon	Single-group longitudinal observational study	Self-efficacy, motivation, somatic anxiety, cognitive anxiety, and physiological performance parameters
Lopez and Sánchez (2023)	Chile; The North Face Endurance Challenge, Santiago de Chile, with 10, 21, 50, 80, and 160 km distances	86 complete mountain/trail runner cases; 60 men and 26 women; 42% ultradistance and 58% shorter trail distances	Cross-sectional observational analytical study	NEO-FFI personality traits, declared race goal, and race placement
Masters and Lambert (1989)	United States; 1987 St. George Marathon, Utah	48 marathon runners; 30 men and 18 women; mean age 33.35 years; average 47.5 training miles per week	Prospective observational race-diary study	Cognitive strategy use, performance time, injury history/dropout, and reasons for running
Masters and Ogles (1998)	Marathon runners recruited during marathon race registration with four-month follow-up in Study 2.	Study 1: 127 marathon runners, 89% male, mean age 38.5 years. Study 2: 188 marathon runners, 78% male, mean age 37.8 years; 156 returned follow-up data.	Two-study observational field report with one retrospective/cross-sectional study and one prospective four-month injury follow-up study.	Running-related injury, race performance time, cognitive strategy preference, running investment, and motivation.
Miller et al. (2023)	Online survey and narrative interviews with elite ultramarathon runners.	61 elite ultramarathon runners in the quantitative phase, including 39 males; seven elite ultramarathon runners in the qualitative phase.	Mixed-methods cross-sectional questionnaire and narrative interview study.	Relative autonomy of sport motivation, composite anxiety symptoms, and qualitative accounts of anxiety and adaptive or maladaptive behavior.
Méndez-Alonso et al. (2021)	Spain; Travesera Integral Picos de Europa ultra-trail race in Picos de Europa National Park.	356 ultra-trail runners from 450 registered participants; 309 men and 47 women; mean age 42.73 years; mean ultra-trail experience 5.7 years; 148 finishers and 208 withdrawals.	Observational race-field study using pre- and post-race questionnaires linked to official race performance.	Race completion, race time or final classification, psychological profile, and pre- to post-race psychological change.
Nicolas et al. (2019)	Ultra-Trail du Mont-Blanc mountain ultra-marathon; Western Europe; route details not fully specified in report	29 mountain ultra-marathon finishers; 17 men and 12 women; age range 28 to 63 years	Six-wave one-month longitudinal observational field study	Sport Emotion Questionnaire anxiety, dejection, anger, happiness, and excitement
Nicolas et al. (2022)	Tor des Géants mountain ultra-marathon, Italy; Western Europe	13 finishers from 17 initially recruited; 11 men and 2 women; mean age 40.08 years	Repeated-measures observational race-field study	Recovery and stress states from the French RestQ-36-R-Sport
Nikolaidis and Knechtle (2018)	Athens Authentic Marathon 2017, Greece; laboratory testing in Nikaia, Greece	156 recreational marathon runners; 26 women and 130 men	Cross-sectional observational analytical study	Pace range and coefficient of variation of race speed; motivation profile
Philippe et al. (2016)	France (Réunion Island); Grand Raid de la Réunion ultra-trail races	Ten amateur ultra-trail runners who withdrew from Diagonale des Fous, Bourbon Trail, or Mascareignes Trail; 8 men and 2 women; mean age 38.3 years.	Qualitative retrospective self-confrontation course-of-experience study	Race withdrawal decision process; pain/discomfort appraisal; coping responses; social influences.
Popov et al. (2019)	Serbia; online endurance running survey	Two hundred eighty-nine runners who had completed at least one mini-marathon, half-marathon, or marathon; 54% male; mean age 36.65 years.	Cross-sectional online psychometric and correlational survey	Positive affect, negative affect, and subjective well-being.
Pruszczak and Stolarski (2025)	Poland; Warsaw 10-km Independence Run and a Polish half-marathon	Four hundred sixty-one total finishers across two studies: 337 10-km runners and 124 half-marathon runners, mostly recreational or amateur athletes.	Two-study prospective race-field survey with official performance outcomes	Official race time performance; mediation through body mass index, subjective preparedness, and sport engagement vigor.
Roebuck et al. (2018)	Melbourne, Australia; laboratory cold pressor pain-testing study	20 ultramarathon runners and 20 age- and gender-matched controls	Matched case–control pilot study	Cold pressor pain tolerance; pain-related anxiety; pain catastrophizing; pain vigilance; pain-specific resilience
Rubaltelli et al. (2018)	Verona, Italy; half-marathon bib pickup and official race timing	237 half-marathon runners; 27% female; mean age 44 years	Pre-race observational field study using structural equation modeling	Official half-marathon finish time; desired and expected finish time; training load context
Jaenes Sánchez et al. (2009)	Spain; XXII Maratón Ciudad de Sevilla runner expo and official marathon results	189 adult popular marathon runners; mean age 39.98 years; 170 men, 9 women, and 10 not reporting sex	Cross-sectional observational race-field questionnaire study	Expected race time, obtained race time, last prior race time, and hardy personality profile
Schüler and Brunner (2009)	Marathon race settings	Three samples of marathon runners: Study 1 *n* = 112, Study 2 *n* = 109, Study 3 *n* = 65 male runners	Three-study observational marathon-runner field report	Future running motivation, pre-race training behavior, and official marathon race time
Tapia-Serrano et al. (2020)	Spain; Behobia-San Sebastián long-distance race	1849 adult long-distance runners aged 18 to 74 years; 1,343 men and 506 women	Cross-sectional correlational mediation study	Perceived health status and expected race outcome
Timm et al. (2017)	United States; 2013 Boston Marathon, Boston, Massachusetts	16 runners who competed in the 2013 Boston Marathon; 7 men and 9 women; mean age 39 years	Retrospective qualitative semi-structured interview study	Resilience process, coping and emotional processing, psychosocial resources, and positive outcomes
Urquijo et al. (2024)	Spain; two official short-distance trail races in the Basque Country	142 trail runners; 119 men and 23 women; mean age 36.11 years	Cross-sectional online questionnaire mediation study	Perceived stress and negative self-talk mediation
van Iperen et al. (2022a)	Netherlands; online survey of recreational long-distance runners	425 recreational long-distance runners training for half-marathon or marathon distances; 57.2% men and 42.8% women; mean age 44.7 years	Cross-sectional latent profile analysis	Running-related injury and chronic fatigue
van Iperen et al. (2022b)	Belgium; Belfius Brussels Marathon 2016 half-marathon and marathon finishers	623 recreational long-distance runners; 498 half-marathon and 125 marathon finishers; mean age 40.0 years; 31.6% women and 67.6% men	Cross-sectional post-event survey with hierarchical regression moderation analyses	Physical strength, cognitive liveliness, and emotional energy dimensions of vigor
de la Vega et al. (2011)	Spain; Trail Aneto 2009 mountain ultradistance race and La Melonera 10.06 km popular race	130 endurance runners: 69 mountain ultradistance runners and 61 10.06 km runners; total mean age 35.4 years	Descriptive cross-sectional comparative field study	Hardy personality profile, ultra-trail completion, and ultra-trail race time
Waleriańczyk and Stolarski (2021)	Poland; Warsaw 10-km Independence Run and a Polish half-marathon	Study 1: 332 10-km finishers; Study 2: 133 recruited half-marathon participants and 115 finishers analyzed; mostly recreational or amateur runners	Two pre-race observational field studies linked to official race results	10-km and half-marathon race performance and anticipated performance
Waleriańczyk (2023)	Poland; European Running Festival mountain trail races	167 Polish trail runners, 54 women and 113 men, aged 19 to 65 years, competing in 20 km, 43 km, 60 km, or 105 km trail races	Prospective observational field study with pre-race personality assessment linked to official race performance	Transformed trail-running performance score based on official race results, Naismith’s rule, and world-record comparison
Woodman and Welch (2022)	Marathon and ultramarathon endurance running context	35 marathon and ultramarathon runners	Single-event observational pre- and post-race moderation study	Pre- to post-race change in anxiety

Table 2 summarizes each study’s psychological exposure or concept, measurement approach or timing, and methodological context. The exposures were diverse and included pain-related coping, general coping and metacognitive self-regulation, mental toughness, self-efficacy, motivation, emotional intelligence, personality dimensions, perfectionism, resilience, pain tolerance, pain-related anxiety, and other trait-like or state-like psychological constructs assessed in distance-running or ultramarathon contexts.

**Table 2:** Methodological characteristics and psychological exposures of the included studies.

Study	Psychological exposure or concept	Measurement, timing, or setting
Alschuler et al. (2019)	Experiential awareness, adaptive pain coping, and maladaptive pain coping were assessed as repeated coping composites using brief stage-level self-report items.	Daily questionnaires completed after race stages; stage-specific deviations and person-mean scores used in multilevel models. Five assessed stages during a 250 km, 6-stage race; the sixth short stage not assessed.
Alschuler et al. (2020)	Experiential awareness, adaptive pain coping, and maladaptive pain coping were assessed as stage-level coping composites and averaged across stages for performance analyses.	Daily questionnaires completed after race stages; average coping levels used in logistic and ordinal performance models. Five assessed stages during a 250 km, 6-stage race; the sixth short stage not assessed; race completion assessed at event end.
Beattie et al. (2025)	Mental toughness, resilience, coping effectiveness, coping frequency, and qualitative coping strategies were assessed as psychological resources or coping processes.	Mental Toughness Inventory, Connor-Davidson Resilience Scale 10-item, Brief Resilience Scale, modified Coping Effectiveness Scale, bespoke coping-frequency item, and open-ended responses. Pre-race trait measures; post-race coping effectiveness and satisfaction; coping frequency retrospectively reported for 11 checkpoint intervals.
Bennett et al. (2026)	Pain narrative and meaning-making; problem-focused pain management and bodily monitoring; chosen pain and agency; relational pain management and collective care.	Ethnographic observations during training camp and event plus three semi-structured interview time points. 5-day training camp observations, 7 event-day observations, and interviews at four months before, one month before, and one month after the race.
Boere et al. (2026)	Psychological distress/negative affect via Depression Anxiety Stress Scales-21 and motivation subtypes via Sport Motivation Scale; contextual 50-km race exposure retained for article completeness.	Online questionnaires 7 to 14 days pre-race; a visual oddball task with electroencephalography before and within 10 min after the race. Acute single 50-km ultramarathon; average race time 7 h 54 min; post-race testing within 10 min.
Brace et al. (2020)	Sports mental toughness and endurance sport self-efficacy assessed before the elite 100-mile ultramarathon competition.	Online survey hosted on SurveyMonkey; official race-performance data obtained from event website. Survey completed within 12 days before race start, mean 6.16 days before race start; HURT100 was a 161-km event.
Brick et al. (2015)	Metacognitive self-regulation and attentional-focus strategies including planning, monitoring, active self-regulation, active distraction/switching off, and reviewing/evaluation.	Measured through semi-structured interviews; strategy use was discussed for training and competition contexts. Retrospective accounts; one interview per participant; interviews lasted 55 to 98 min, mean 75.5 min.
Buman et al. (2008)	Coping responses to hitting the wall, including cognitive strategies, emotion-focused coping, physical race-management efforts, and lack of strategy.	Measured with web-based open-ended questions and grounded theory coding; no standard definition of hitting the wall was imposed. A retrospective account of marathon hitting-the-wall episodes; participants completed the web study in 10 to 30 min.
Chalabaev et al. (2017)	Self-determined motivation, autonomous motivation, controlled motivation, external regulation, and amotivation toward marathon running.	Measured the day before the race using Sports Motivation Scale in Study 1 and Situational Motivation Scale in Study 2; injury-risk perceptions measured by four-item susceptibility scale and one pain-continuity risk item in Study 2. A single pre-race questionnaire assessment; Study 1 *n* = 378 and Study 2 *n* = 339.
Christensen et al. (2018)	Mental toughness, mindfulness, pain catastrophizing, and attentional focus/cognitive orientation during a typical ultramarathon.	Measured using the Sports Mental Toughness Questionnaire, State Mindfulness Scale, Pain Catastrophizing Scale, and Cognitive Orientation Classification System through online/on-site questionnaire. Single assessment before or around the 2014 Western States Endurance Run; survey duration approximately 15 to 20 min.
Corrion et al. (2018)	Self-efficacy, intention to finish, mastery-approach goals, motivation, and coping strategies including seeking social support and avoidance coping.	Measured with adapted the Sport Motivation Scale II, theory of planned behavior items, achievement-goal questionnaire, basic needs scale, and Brief COPE-based coping items. Single pre-race questionnaire one day before the 140-km ultra-trail race; dropout determined after the event.
Deaner et al. (2019)	Pace-related risk-taking, willingness to suffer during the marathon, competitiveness, personal goal achievement, and domain-specific risk-taking.	Measured through a Qualtrics online survey using newly developed pace-related risk-taking and WSM scales, abbreviated Motivations of Marathoners Scales subscales, and abbreviated DOSPERT items. A single retrospective survey addressed the most recent best-effort marathon; pacing was calculated from self-reported first- and second-half times.
Diotaiuti et al. (2021)	Autonomy satisfaction, competence satisfaction, locomotion mode, homeostatic reintegration, and resilient reintegration were modeled as psychological need, self-regulatory, and resilience-process constructs.	Online questionnaire with validated Italian adaptations of the Basic Psychological Need Satisfaction Scale, Self-Regulatory Modes Scale, and Resilience Process Questionnaire. Single cross-sectional assessment; no intervention dose or follow-up.
Freund et al. (2013)	Extreme ultramarathon runner status, personality dimensions, and general self-efficacy were compared with matched non-marathon controls and related to cold pain tolerance.	Cold pressor test below 2 °C for up to 3 min; Trait and Character Inventory; General Self-Efficacy Scale. Single testing session; runners tested 12 to 18 days before the TransEurope FootRace; controls tested during the following three months.
Gameiro et al. (2023)	Mental toughness and resilience were assessed as psychological resources in trail runners.	Sport Mental Toughness Questionnaire, Connor-Davidson Resilience Scale 10-item, and International Trail Running Association Performance Index. Single cross-sectional assessment; performance index based on certified trail races in the previous 36 months.
Gauld et al. (2024)	Exercise addiction symptoms (EAI) and Big Five personality dimensions (TIPI).	Self-administered EAI and TIPI combined with medical-file and phone-interview data. Retrospective/post-complication assessment; EAI cut-offs and symptom-network analysis used; TIPI compared with normative scores.
Gayton et al. (1986)	Physical self-efficacy, perceived physical ability, and physical self-presentation confidence.	Self-report scale administered before the marathon; predicted time recorded; actual time from official posted results. Single pre-race assessment shortly before race start; actual outcome same day.
Gillet et al. (2012)	Self-determination theory motivation forms and derived motivational profiles.	Online pre-race Sport Motivation Scale; hierarchical and k-means cluster analyses derived three profiles. Single pre-race survey; race performance measured by official final ranking after the Marathon des Sables.
Graham et al. (2021)	Mental toughness measured with MT18 and race sleep quantity measured by self-assessment sleep log.	MT18 administered pre-event; injuries assessed clinically each day; mood with Brunel Mood Scale; sleep from post-event self-assessment log. Three-day 120-mile Arctic race; daily/event injury and mood assessment; mean sleep 4.1 ± 2.8 h.
Gross (2025)	Pain and suffering narratives, self-talk, and moral language were characterized through interviews, blogs, and participant observation.	Semi-structured interviews, approximately 20 blogs, and fieldnotes were analyzed with reflexive thematic analysis. Retrospective accounts across prior ultraraces; interviews lasted approximately 30 min to 2 h; participant observation across events.
Howe et al. (2019)	Trait emotional intelligence assessed as a pre-race psychological trait and grouped using a median split.	33-item trait EI scale; Brunel Mood Scale, serum cortisol, RPE, and completion time measured during controlled treadmill ultramarathon. Trait EI within two weeks before trial; 80.5 km treadmill race completed in 09:00:18 ± 01:14:07 on average.
Jackman et al. (2024)	Mental contrasting with implementation intentions, goal revision, and goal disengagement/re-engagement as self-regulatory processes during goal striving.	Event-focused interviews reconstructed race timelines and used time-ordered matrix analysis of desired future, present reality, goal-attainment expectancy, and decisions. Runners were interviewed a mean 77.43 h after excellent competitive performances; processes were reconstructed across self-identified race stages.
Jaenes et al. (2022)	Psychological wellbeing dimensions: self-acceptance, autonomy, environmental mastery, purpose in life, personal growth, and positive relations.	Spanish-adapted reduced 20-item Psychological Wellbeing Scale; competitive anxiety measured using Spanish-adapted Sport Anxiety Scale-2. Questionnaires completed 24 h before the Seville Marathon at the race expo.
Jowett et al. (2018)	Self-oriented perfectionism, socially prescribed perfectionism, and derived 2 × 2 perfectionism subtypes.	Multidimensional Perfectionism Scale Short Form prefaced with ‘In my sport’; Brief COPE adapted to injury context for coping outcomes. Single hard-copy or online questionnaire; injury history covered the previous 12 months.
Kelemen et al. (2025)	Personality traits, motivational styles, self-confidence, and mental preparation variables.	Single assessment; precompetition anxiety/profile measures.
Kilduff (2014)	Interindividual rivalry as a relational competitive-motivation construct.	Rivals were inferred from archival race histories using similarity, repeated competition, and evenly matched prior contests; a pilot survey supported rivalry as common and motivational among runners. Rivals identified from 2004 to 2006 races and tested against 2007 to 2009 race performance.
Krokosz et al. (2018)	Personality traits and motives for ultramarathon participation.	EPQ-R assessed personality traits; IPAO assessed 12 participation objectives. Extracted results focused on extraversion, neuroticism, company of other people, managing stress, and escape from everyday life. Measured 12 h before the 100 km run, with mood measured 12 h before and 12 h after the race.
Lane and Wilson (2011)	Trait emotional intelligence.	33-item Emotional Intelligence Scale; high and low trait emotional intelligence groups formed by median split. Emotional states measured using Brunel Mood Scale plus UWIST calmness and happiness items. Trait emotional intelligence measured at registration before the event; mood measured at breakfast and after each of six stages.
Larumbe-Zabala et al. (2020)	Perceived physical fitness, self-efficacy, motivation, somatic anxiety, cognitive anxiety, and perceived social support.	Podium questionnaire visual analogue scales scored from 0 to 100; physiological tests included ventilatory threshold speeds and running economy but were contextual rather than psychological exposures. Repeated at five pre-marathon timepoints across a 16-week training macrocycle.
Lopez and Sánchez (2023)	Big Five personality traits and declared race goal or motivation category.	NEO Five-Factor Inventory plus study-specific online sport-practice and motivation questions; race results used for placement outcomes. Single online questionnaire linked to one Chilean trail/ultra-trail race result.
Masters and Lambert (1989)	Association and dissociation cognitive strategies, and reasons for running a marathon with emphasis on drive/competition.	Marathon Race Diary coded by blinded raters using Schomer’s system; Masters Reasons for Running a Marathon Scale rated 29 reasons on a 1 to 7 scale. Race diary completed within 24 h after the marathon; reasons-for-running assessed as a trait-like motivation measure.
Masters and Ogles (1998)	Association and dissociation cognitive strategies, competitive/goal marathon motivation, and running investment/addiction.	Study 1 used percentage estimates of time spent associating/dissociating during training and the marathon plus MOMS; Study 2 used the Attentional Focus Questionnaire, MOMS, Sport Orientation Questionnaire, and Running Addiction Scale. Study 1 assessed previous-year injury and race-related strategy. Study 2 assessed typical training attentional focus before a four-month injury follow-up.
Miller et al. (2023)	Irrational performance beliefs and motivation regulation/relative autonomy in elite ultramarathon runners.	Qualtrics survey using iPBI-II, SMS-II Relative Autonomy Index, and SAS-II, followed by narrative interviews with seven runners. Cross-sectional survey and qualitative interviews; no intervention or follow-up.
Méndez-Alonso et al. (2021)	Mental toughness, psychological resilience, harmonious passion, and obsessive passion.	Online self-report questionnaires: 7-item Mental Toughness Inventory, Spanish 14-item Resilience Scale, and passion questionnaire. Pre-race questionnaire in the week before the ultra-trail race and post-race questionnaire in the week after the race.
Nicolas et al. (2019)	Trait emotional intelligence and its appraisal, regulation, and utilization dimensions.	Brief Emotional Intelligence Scale 10-item version; Sport Emotion Questionnaire for post-race emotions. Emotional intelligence assessed within two days before the race; emotions assessed within 2 h after finish and at 7, 14, 21, and 28 days.
Nicolas et al. (2022)	Trait emotional intelligence grouped as high versus low using a median split.	Brief Emotional Intelligence Scale 10-item version and French Recovery-Stress Questionnaire for Athletes RestQ-36-R-Sport. Trait emotional intelligence measured before the race; recovery-stress states assessed pre-race, mid-race after 155 km, and within 3 h after finishing.
Nikolaidis and Knechtle (2018)	Marathon motivation dimensions: psychological coping, self-esteem, life meaning, health orientation, weight concern, affiliation, recognition, competition, and goal achievement.	Motivations of Marathoners Scales, 56 items, 7-point response scale. Motivation questionnaire and physiological tests performed about one month before the Athens Authentic Marathon; pacing extracted from official split times.
Philippe et al. (2016)	Withdrawal decision-making and coping process; pain-related meaning-making; other-runner and support-person influences.	Self-confrontation interviews, race-map traces, and course-of-action coding of Elementary Units of Meaning and representative sequences. Race experiences were reconstructed 1 to 3 days after withdrawal; sequences covered pain/discomfort, meaning-making, running-style adjustment, coping attempts, social influence, situational assessment, and withdrawal.
Popov et al. (2019)	Motivation dimensions: Mental Health Improvement, Stress Coping, Affiliation, Physical Health and Condition, Competitive Spirit, and Physical Appearance.	Modified Serbian Motivations of Marathoners Scale with exploratory six-factor structure; positive and negative affect assessed with Serbian PANAS adaptation; subjective well-being with Short Subjective Well-Being Scale. Cross-sectional online assessment after prior endurance running race participation; no target race timepoint.
Pruszczak and Stolarski (2025)	Sport time perspectives, especially Future-Positive, Future-Negative, and Present-Fatalistic; sport engagement/vigor and subjective preparedness in Study 2.	Sport Time Perspective Questionnaire; Sport Engagement Scale; self-rated preparedness; official race results. Questionnaires were available from two days before the race until race start; performance obtained from official results.
Roebuck et al. (2018)	Pain-related anxiety symptoms, pain-related escape/avoidance, pain catastrophizing, pain vigilance/awareness, and pain-specific resilience.	PASS-20, PCS, PVAQ, and PRS self-report measures completed before cold pressor testing. Single laboratory session; cold pressor test performed after questionnaires.
Rubaltelli et al. (2018)	Trait emotional intelligence and pre-race performance aspiration/expectancy.	TEIQue-SF and items assessing desired and expected half-marathon time; official timing data linked by name or bib number. Questionnaire completed the day before the half-marathon; official finish time obtained after the race.
Jaenes Sánchez et al. (2009)	Hardy personality total and dimensions: control, commitment, and challenge.	Escala de Personalidad Resistente en Maratonianos, 30 items, with scores transformed to 0 to 100; performance comparisons used a median split of total score. Assessed 36 to 12 h before the Seville Marathon at the runner expo.
Schüler and Brunner (2009)	Flow experience during marathon race and during training.	Flow Short Scale; race flow assessed at kilometer 10, 20, 30, and 40; Studies 1 and 2 used retrospective ratings, Study 3 used in-race experience sampling. Race-segment flow during the marathon; training flow for a typical training situation in the preceding 10 weeks excluding the final week.
Tapia-Serrano et al. (2020)	Intrinsic motivation, extrinsic motivation, and expected race performance.	Behavioural Regulation in Exercise Questionnaire-3 for motivation; expected race outcome reported as expected finishing time; perceived health assessed with a single item. Cross-sectional questionnaire administered electronically through race organizers.
Timm et al. (2017)	Post-traumatic sport resilience process, cognitive and behavioral coping, and psychosocial resources after a traumatic marathon event.	Semi-structured interviews using questions guided by Galli and Vealey’s sport resilience model; thematic analysis of race-day adversity, coping, resources, and outcomes. Interviews were conducted 10 to 12 months after the 2013 Boston Marathon bombings.
Urquijo et al. (2024)	Emotion regulation and negative self-talk as antecedents or mediators of perceived stress.	WLEIS emotion regulation subscale, Automatic Self-Talk Questionnaire for Sports negative self-talk subscale, and Perceived Stress Scale short form. Online questionnaire completed up to one week before a trail race; negative self-talk referenced recent months in sport.
van Iperen et al. (2022a)	Passion for running, running-related resources, and running-related recovery combined into latent psychological risk profiles.	Dutch Vallerand Passion Scale; DISQ-SPORT resources; DISQ-R SPORT recovery; latent profile analysis. Cross-sectional online questionnaire in 2018; running-related injuries recalled over the prior 12 months.
van Iperen et al. (2022b)	Running-related demands, resources, and recovery were assessed in physical, cognitive, and emotional dimensions.	DISQ-Sport measured demands/resources and DISQ-R Sport measured detachment/recovery; vigor was measured with the Shirom-Melamed Vigor Measure. Recovery referenced the week before the Brussels Marathon and vigor referenced the week after the event; no intervention dose.
de la Vega et al. (2011)	Hardy personality total, commitment, control, and challenge were measured as resilience-related personality constructs.	Adapted Escala de Personalidad Resistente en Maratonianos administered to mountain ultradistance and 10.06 km runners. Single race-day assessment before or near competition; Trail Aneto race was 78 km with 3,500 m positive elevation.
Waleriańczyk and Stolarski (2021)	Perfectionistic strivings, perfectionistic concerns, and Big Five personality traits were assessed as personality exposures.	Short Perfectionism in Sport Questionnaire in both studies; IPIP-BFM-20 Big Five traits in Study 2. Online pre-race questionnaire, ranging from days to minutes before the race; official race results linked after competition.
Waleriańczyk (2023)	Perfectionistic strivings, perfectionistic concerns, and derived 2 × 2 perfectionism subtypes or tipping points were treated as personality-dimension exposures.	Measured using sport-specific perfectionism instruments: Performance Perfectionism Scale-Sport and Sport Multidimensional Perfectionism Scale-2; subscales were standardized and aggregated into higher-order dimensions. Questionnaire completed in the week before the trail-running competition; official performance imported after the race.
Woodman and Welch (2022)	Alexithymia was treated as a trait-like emotion-processing exposure mapped to the emotional intelligence/emotion regulation family.	Alexithymia and pre- and post-race anxiety were assessed around marathon or ultramarathon participation; exact timing was not reported in the source article.

### 3.3 Risk of bias and methodological quality within included evidence units

Critical appraisal was conducted using Joanna Briggs Institute checklists matched to each study design. The 53 included studies generated 55 checklist records because Masters and Ogles (1998) were appraised with both the cohort and analytical cross-sectional checklists, and Miller et al. (2023) were appraised with both the analytical cross-sectional and qualitative checklists. Methodological quality assessments indicated minor methodological concerns in 3 studies, moderate concerns in 36, and serious concerns in 14. Overall judgments for individual studies are presented in Table 3.

**Table 3:** Overall critical appraisal judgments for the included studies.

Article	Appraisal tool used	Overall judgment
Alschuler et al. (2019)	JBI Critical Appraisal Checklist for Cohort Studies	Moderate risk/methodological concerns
Alschuler et al. (2020)	JBI Critical Appraisal Checklist for Cohort Studies	Moderate risk/methodological concerns
Beattie et al. (2025)	JBI Critical Appraisal Checklist for Cohort Studies	High risk/serious methodological concerns
Bennett et al. (2026)	JBI Critical Appraisal Checklist for Qualitative Research	Low risk/minor methodological concerns
Boere et al. (2026)	JBI Critical Appraisal Checklist for Cohort Studies	Moderate risk/methodological concerns
Brace et al. (2020)	JBI Critical Appraisal Checklist for Cohort Studies	Moderate risk/methodological concerns
Brick et al. (2015)	JBI Critical Appraisal Checklist for Qualitative Research	Moderate risk/methodological concerns
Buman et al. (2008)	JBI Critical Appraisal Checklist for Qualitative Research	Moderate risk/methodological concerns
Chalabaev et al. (2017)	JBI Critical Appraisal Checklist for Analytical Cross-Sectional Studies	High risk/serious methodological concerns
Christensen et al. (2018)	JBI Critical Appraisal Checklist for Cohort Studies	High risk/serious methodological concerns
Corrion et al. (2018)	JBI Critical Appraisal Checklist for Cohort Studies	Moderate risk/methodological concerns
Deaner et al. (2019)	JBI Critical Appraisal Checklist for Analytical Cross-Sectional Studies	Moderate risk/methodological concerns
Diotaiuti et al. (2021)	JBI Critical Appraisal Checklist for Analytical Cross-Sectional Studies	Moderate risk/methodological concerns
Freund et al. (2013)	JBI Critical Appraisal Checklist for Analytical Cross-Sectional Studies	High risk/serious methodological concerns
Gameiro et al. (2023)	JBI Critical Appraisal Checklist for Analytical Cross-Sectional Studies	Moderate risk/methodological concerns
Gauld et al. (2024)	JBI Critical Appraisal Checklist for Analytical Cross-Sectional Studies	High risk/serious methodological concerns
Gayton et al. (1986)	JBI Critical Appraisal Checklist for Cohort Studies	High risk/serious methodological concerns
Gillet et al. (2012)	JBI Critical Appraisal Checklist for Cohort Studies	Moderate risk/methodological concerns
Graham et al. (2021)	JBI Critical Appraisal Checklist for Cohort Studies	High risk/serious methodological concerns
Gross (2025)	JBI Critical Appraisal Checklist for Qualitative Research	Low risk/minor methodological concerns
Howe et al. (2019)	JBI Critical Appraisal Checklist for Cohort Studies	High risk/serious methodological concerns
Jackman et al. (2024)	JBI Critical Appraisal Checklist for Qualitative Research	Low risk/minor methodological concerns
Jaenes et al. (2022)	JBI Critical Appraisal Checklist for Analytical Cross-Sectional Studies	Moderate risk/methodological concerns
Jowett et al. (2018)	JBI Critical Appraisal Checklist for Analytical Cross-Sectional Studies	Moderate risk/methodological concerns
Kelemen et al. (2025)	JBI Critical Appraisal Checklist for Analytical Cross-Sectional Studies	High risk/serious methodological concerns
Kilduff (2014)	JBI Critical Appraisal Checklist for Cohort Studies	Moderate risk/methodological concerns
Krokosz et al. (2018)	JBI Critical Appraisal Checklist for Cohort Studies	High risk/serious methodological concerns
Lane and Wilson (2011)	JBI Critical Appraisal Checklist for Cohort Studies	Moderate risk/methodological concerns
Larumbe-Zabala et al. (2020)	JBI Critical Appraisal Checklist for Cohort Studies	High risk/serious methodological concerns
Lopez and Sánchez (2023)	JBI Critical Appraisal Checklist for Analytical Cross-Sectional Studies	Moderate risk/methodological concerns
Masters and Lambert (1989)	JBI Critical Appraisal Checklist for Analytical Cross-Sectional Studies	Moderate risk/methodological concerns
Masters and Ogles (1998)	JBI Critical Appraisal Checklist for Analytical Cross-Sectional Studies; JBI Critical Appraisal Checklist for Cohort Studies	Moderate risk/methodological concerns
Miller et al. (2023)	JBI Critical Appraisal Checklist for Analytical Cross-Sectional Studies; JBI Critical Appraisal Checklist for Qualitative Research	Moderate risk/methodological concerns
Méndez-Alonso et al. (2021)	JBI Critical Appraisal Checklist for Cohort Studies	Moderate risk/methodological concerns
Nicolas et al. (2019)	JBI Critical Appraisal Checklist for Cohort Studies	Moderate risk/methodological concerns
Nicolas et al. (2022)	JBI Critical Appraisal Checklist for Cohort Studies	High risk/serious methodological concerns
Nikolaidis and Knechtle (2018)	JBI Critical Appraisal Checklist for Analytical Cross-Sectional Studies	Moderate risk/methodological concerns
Philippe et al. (2016)	JBI Critical Appraisal Checklist for Qualitative Research	Moderate risk/methodological concerns
Popov et al. (2019)	JBI Critical Appraisal Checklist for Analytical Cross-Sectional Studies	Moderate risk/methodological concerns
Pruszczak and Stolarski (2025)	JBI Critical Appraisal Checklist for Analytical Cross-Sectional Studies	Moderate risk/methodological concerns
Roebuck et al. (2018)	JBI Critical Appraisal Checklist for Analytical Cross-Sectional Studies	High risk/serious methodological concerns
Rubaltelli et al. (2018)	JBI Critical Appraisal Checklist for Cohort Studies	Moderate risk/methodological concerns
Jaenes Sánchez et al. (2009)	JBI Critical Appraisal Checklist for Analytical Cross-Sectional Studies	Moderate risk/methodological concerns
Schüler and Brunner (2009)	JBI Critical Appraisal Checklist for Cohort Studies	Moderate risk/methodological concerns
Tapia-Serrano et al. (2020)	JBI Critical Appraisal Checklist for Analytical Cross-Sectional Studies	Moderate risk/methodological concerns
Timm et al. (2017)	JBI Critical Appraisal Checklist for Qualitative Research	Moderate risk/methodological concerns
Urquijo et al. (2024)	JBI Critical Appraisal Checklist for Analytical Cross-Sectional Studies	Moderate risk/methodological concerns
van Iperen et al. (2022a)	JBI Critical Appraisal Checklist for Analytical Cross-Sectional Studies	Moderate risk/methodological concerns
van Iperen et al. (2022b)	JBI Critical Appraisal Checklist for Analytical Cross-Sectional Studies	Moderate risk/methodological concerns
de la Vega et al. (2011)	JBI Critical Appraisal Checklist for Analytical Cross-Sectional Studies	Moderate risk/methodological concerns
Waleriańczyk and Stolarski (2021)	JBI Critical Appraisal Checklist for Analytical Cross-Sectional Studies	Moderate risk/methodological concerns
Waleriańczyk (2023)	JBI Critical Appraisal Checklist for Analytical Cross-Sectional Studies	Moderate risk/methodological concerns
Woodman and Welch (2022)	JBI Critical Appraisal Checklist for Cohort Studies	High risk/serious methodological concerns

The sensitivity synthesis excluded the 14 studies classified as having serious methodological concerns and retained 39 studies: 3 with minor concerns and 36 with moderate concerns. This restriction did not change the principal finding that maladaptive pain coping was associated with greater pain interference and a lower likelihood of race completion, as these associations were supported by the repeated-measures studies of Alschuler et al. (2019, 2020), both of which had moderate methodological concerns.

The general association of emotional intelligence or emotion regulation with mood- and stress-related outcomes was also retained. Although the exclusion removed evidence from Howe et al. (2019) and Nicolas et al. (2022), related associations remained in Lane and Wilson (2011), Nicolas et al. (2019), and Urquijo et al. (2024). The restricted evidence, therefore, continued to support an association with affective and perceived stress outcomes, but evidence concerning cortisol responses and recovery resources became narrower and should be interpreted with greater caution (Table 4).

**Table 4:** Sensitivity table.

Principal synthesis finding	Studies in full synthesis	Serious-concern studies excluded	Evidence remaining	Sensitivity classification	Revised interpretation
Maladaptive coping and pain interference/completion	Alschuler et al. (2019, 2020)	None	Both studies retained	Retained	Association remains supported, with moderate concerns
Emotional intelligence/emotion regulation and mood/stress	Howe et al. (2019); Lane and Wilson (2011); Nicolas et al. (2019, 2022); Urquijo et al. (2024)	Howe et al. (2019); Nicolas et al. (2022)	Lane and Wilson (2011); Nicolas et al. (2019); Urquijo et al. (2024)	Retained but attenuated	Affective/stress association remains; physiological-recovery evidence weakened
Mental toughness/resilience/self-efficacy and performance	Multiple studies	Beattie et al. (2025); Christensen et al. (2018); Graham et al. (2021)	Brace et al. (2020); Gameiro et al. (2023); de la Vega et al. (2011)	Retained	Associations remain heterogeneous and non-deterministic
Pain tolerance and pain-related anxiety	Freund et al. (2013); Roebuck et al. (2018)	Both studies	No comparable evidence	Not robust	Remove from principal conclusions
Motivation and continuation through pain	Chalabaev et al. (2017)	Chalabaev et al. (2017)	No direct comparable evidence	Not robust	Treat as preliminary single-study evidence
Exercise addiction symptoms and serious medical complications	Gauld et al. (2024)	Gauld et al. (2024)	No comparable evidence	Not robust	Treat as rare-event exploratory evidence
Alexithymia and anxiety regulation	Woodman and Welch (2022)	Woodman and Welch (2022)	No comparable evidence	Not robust	Treat as preliminary
Physical self-efficacy and marathon time	Gayton et al. (1986)	Gayton et al. (1986)	No direct comparable evidence	Not robust	Do not retain as a general conclusion

The conclusion that mental toughness, resilience, and self-efficacy show heterogeneous and context-dependent associations with performance was retained. The exclusion of Beattie et al. (2025), Christensen et al. (2018), and Graham et al. (2021) reduced the evidence concerning coping–performance satisfaction interactions, psychological prediction in individual ultramarathons, and mental toughness in relation to mood and injury. However, mixed positive and null associations remained across Brace et al. (2020), Corrion et al. (2018), Gameiro et al. (2023), Méndez-Alonso et al. (2021), and de la Vega et al. (2011). Thus, the restricted synthesis continued to argue against a uniform or deterministic relationship between resilience-related traits and objective performance.

Several secondary findings were not robust to the exclusion. The comparative evidence that ultramarathon runners have greater cold-pain tolerance and lower pain-related anxiety was derived from Freund et al. (2013) and Roebuck et al. (2018), both of which had serious methodological concerns. The association between motivational regulation and the willingness to continue running through pain depended on Chalabaev et al. (2017). Evidence concerning exercise addiction symptoms among runners with serious medical complications and alexithymia-related anxiety regulation depended on Gauld et al. (2024) and Woodman and Welch (2022), respectively. Similarly, selected associations involving physical self-efficacy and marathon time, mindfulness intervention responses, pre-race personality and mood, and psychological state in relation to running speed were no longer represented after the exclusion of Gayton et al. (1986), Kelemen et al. (2025), Krokosz et al. (2018), and Larumbe-Zabala et al. (2020). Overall, the restricted synthesis preserved the three central conclusions: maladaptive coping was associated with poorer pain adaptation and completion outcomes; emotion-regulation constructs were associated primarily with mood and perceived stress outcomes; and associations between resilience-related traits and objective performance were heterogeneous.

### 3.4 Results of individual studies

Individual-study results are grouped by outcome domain in Tables 5–9. Table 5 summarizes studies in the pain adaptation and perceived exertion domain.

**Table 5:** Findings of individual studies in the pain adaptation and perceived exertion domain.

Study	Main comparison or exposure–outcome focus	Main statistical findings	Principal finding
Alschuler et al. (2019)	Within-person and between-person associations between pain coping composites and percent time thinking about pain or pain interference during multistage ultramarathon stages.	Maladaptive pain coping and percent time thinking about pain: between-person beta 6.11, standard error 1.25, *p* < 0.0001; within-person beta 5.56, standard error 1.08, *p* < 0.0001. Maladaptive pain coping and pain interference: between-person beta 0.59, standard error 0.10, *p* < 0.0001; within-person beta 0.70, standard error 0.08, *p* < 0.0001. Experiential awareness and pain interference: between-person beta -0.25, *p* = 0.005; within-person beta -0.22, *p* = 0.013.	Ultramarathon runners used more adaptive than maladaptive coping overall, but higher maladaptive pain coping was associated with more time thinking about pain and greater pain interference. Experiential awareness was associated with lower pain interference.
Bennett et al. (2026)	Qualitative comparison of women’s pain stories across pre-race, early-race, mid-race, late-race, and post-race phases of a six-day ultramarathon.	Themes were generated through thematic narrative and structural narrative analyses of 12 days of ethnographic observations and three semi-structured interview time points with seven women ultrarunners.	Pain was first framed as a problem to solve through monitoring and tangible support. By the midpoint, fatigue and isolation made task-oriented coping insufficient; athletes reframed pain as chosen and drew on collective care, allowing pain to become shared, relational, and empowering.
Freund et al. (2013)	Comparison of TransEurope FootRace ultramarathon runners versus matched controls and correlations of personality/self-efficacy with cold pain tolerance	Cold pressor group difference *p* = 0.0002; time by group *p* < 0.00001; withdrawal latency 180 ± 0 s for runners versus 96 ± 58 s for controls, *p* = 0.0007. GSE 31.5 ± 3.6 in runners versus 29.7 ± 3.3 in controls, not significant. Personality subscale correlations with 180-s pain score included dependence *r* = 0.64 and pure-hearted conscience *r* = 0.66 after conservative correction; runner-only pure-hearted conscience *r* = 0.65, *p* = 0.03.	Extreme ultramarathon runners had markedly greater cold pain tolerance than controls and differed in personality profile; general self-efficacy was not associated with pain tolerance.
Gross (2025)	Reflexive thematic analysis of pain and suffering narratives and self-talk in recreational ultrarunning.	Moral language was generated as the central organizing concept; themes included empowerment, curiosity, glorification, self-image, disciplined body, mental techniques, bodily techniques, verbalized inner speech, did-not-finish, and altered states of perception.	Pain and suffering in ultrarunning were framed as moral language that helps runners justify and cope with negative bodily sensations by connecting them to values such as self-discipline, perseverance, success, and selfhood.
Roebuck et al. (2018)	Ultramarathon runners compared with matched controls; pain-related psychological factors tested as mediators of cold pressor pain tolerance.	Cold pressor immersion: median 180 (0) vs. 119 (141) seconds, z = -3.02, *p* = 0.007, *r* = 0.48. PASS-20 subscales all lower in ultrarunners, *p* ≤ 0.030. Escape/avoidance predicting immersion time after group covariate: b = -4.36, standard erro*r* = 1.78, *p* = 0.020; bootstrap indirect effect b = 0.21, standard erro*r* = 0.10, 95% confidence interval 0.02 to 0.42; approximately 40% mediated.	Ultrarunners had greater cold pain tolerance and lower pain-related anxiety; reduced escape/avoidance partially mediated supranormal pain tolerance.

**Table 6:** Findings of individual studies in the cognitive, attentional, and self-regulatory processes domain.

Study	Main comparison or exposure–outcome focus	Main statistical findings	Principal finding
Boere et al. (2026)	Pre-race versus post-race behavioral and electroencephalography executive-function indices; correlations between prerace motivation/distress and executive-function changes.	Reaction time: 412.2 ± 33.98 ms to 394.1 ± 34.59 ms, t (75 = 5.84, *p* < 0.0001. Reaction-time variability: 50.4 ± 7.65 ms to 57.5 ± 10.89 ms, t (75 = 5.06, *p* < 0.0001. Accuracy: *p* = 0.093. N2 amplitude: 5.3 ± 2.8 μV to 3.8 ± 2.3 μV, results text *p* < 0.0001; abstract *p* = 0.008. P3 amplitude: 6.7 ± 4.0 μV to 5.5 ± 3.5 μV, results text *p* = 0.007; abstract *p* < 0.0001. Identified motivation with N2 reduction *r* = 0.29, *p* = 0.013; introjected motivation with N2 reduction *r* = 0.29, *p* = 0.012; Depression Anxiety Stress Scales-21 with P3 change *r* = -0.69, *p* < 0.001.	A 50-km ultramarathon was followed by reduced N2 and P3 amplitudes and shorter but more variable responses. Prerace motivation and psychological distress were associated with larger neural reductions, suggesting psychological factors may influence cognitive resilience under race strain.
Brick et al. (2015)	Qualitative thematic analysis of metacognitive processes and attentional-focus strategy use in elite endurance runners.	*n* = 10; 6 women and 4 men; interview length 55-98 min, mean 75.5 min. Reports strategy/process participant counts including monitoring bodily sensations *n* = 10, pacing/tactical decisions *n* = 10, relaxation *n* = 10, chunking *n* = 10, knowing when to apply a strategy *n* = 10, and judgments of effective cognitive strategies *n* = 10.	Metacognitive planning, monitoring, reviewing/evaluating, feelings, and judgments were central to cognitive control. Pain and exertion were used as cues for selecting strategies to maintain performance or regulate discomfort.
Buman et al. (2008)	Qualitative analysis of characteristics and coping responses to hitting the wall in recreational marathon runners.	*n* = 57; 40 men and 17 women; mean age 41.79 years. Participants reported mean 4.20 hitting-the-wall characteristics and 2.55 coping strategies. Cognitive strategies *n* = 29 (51%; emotion-focused coping *n* = 7 (12%); race-related physical efforts *n* = 25 (44%); no strategies *n* = 17 (30%).	Hitting the wall was multidimensional. Runners coped through cognitive strategies, physical/behavioral efforts, emotion-focused coping, and willpower, but many reported limited or ineffective coping strategies.
Deaner et al. (2019)	Regression prediction of marathon second-half slowing from risk taking in pace, willingness to suffer, competitiveness, goal achievement, and domain-specific risk taking.	Risk taking in pacing fully adjusted coefficien*t* = 0.728, standard erro*r* = 0.0828, *p* < 2e-16; R^2^ = 0.256; 75th versus 25th quantile predicted difference = 3.64 percentage points more slowing. WSM fully adjusted coefficien*t* = -0.0130, *p* = 0.841. Competitiveness coefficien*t* = 0.106, *p* = 0.187. Goal achievement coefficien*t* = 0.191, *p* = 0.0565. DOSPERT coefficien*t* = -0.0703, *p* = 0.0664.	Pace-related risk-taking was the only robust psychological predictor of greater marathon slowing after full adjustment; willingness to suffer, competitiveness, goal achievement, and domain-specific risk-taking were not robust adjusted predictors.
Jackman et al. (2024)	Qualitative comparison of goal-striving decisions across negative, no, and positive goal-performance discrepancy contexts during excellent distance-running performances.	All 21 runners reported goal persistence at some point; 10 also reported goal disengagement and re-engagement with an alternative. Event-focused interviews averaged 76.04 min and occurred mean 77.43 h post-race.	Mental contrasting with implementation intentions was interpreted as helping runners decide whether to persist, revise goals, or disengage and re-engage with alternative goals. Goal revision appeared adaptive for maintaining effort and averting or managing action crises.
Jowett et al. (2018)	The 2 × 2 perfectionism subtypes and self-oriented or socially prescribed perfectionism as predictors of coping strategies for running injuries.	Problem-focused coping: F(3,209 = 5.39, *p* = 0.01, R2 = 0.048; SOP beta = 0.15, *p* = 0.04; SPP beta = -0.23, *p* < 0.01. Emotion-focused coping: *F*(3,209) = 3.82, *p* = 0.02, R2 = 0.035; SOP beta = 0.15, *p* = 0.04; SPP *p* = 0.38. Avoidance coping: F(3,209) = 7.78, *p* < 0.01, R2 = 0.069; SPP beta = 0.24, *p* < 0.01; SOP *p* = 0.51. Predicted values: problem-focused pure SOP 3.32, non-perfectionism 3.17, mixed 3.10, pure SPP 2.95; emotion-focused pure SOP 2.67 versus non-perfectionism 2.52; avoidance pure SPP 1.72 and mixed 1.76 versus non-perfectionism 1.54.	The 2 × 2 model was supported for problem-focused coping: pure self-oriented perfectionism showed the most adaptive profile, whereas pure socially prescribed perfectionism showed the least adaptive profile. Findings for emotion-focused and avoidance coping were more limited or inconclusive across subtypes.
Masters and Lambert (1989)	Association and dissociation cognitive strategies, reasons for running, injury, and performance time in actual marathon runners.	Drive/competition factor correlated with performance time r(46 = -0.51, *p* < 0.001 and association r(46) = 0.31, *p* < 0.05. Total association mean 74.50, standard deviation 13.98 versus dissociation mean 25.90, standard deviation 14.08; *t* = 12.00, *p* < 0.001. Association correlated with faster performance time r(46) = -0.30, *p* < 0.05. Prior injury dissociation comparison t(46) = 0.54, *p* = 0.59; training-run injury by cognitive strategy χ^2^(2, *N* = 47) = 0.50, *p* > 0.05.	Runners preferred association during the marathon, and association and drive/competition were associated with faster performance, while dissociation was not associated with injury indicators.
Nikolaidis and Knechtle (2018)	Associations of marathon motivation dimensions with pacing range and coefficient of variation in the Athens Authentic Marathon.	Women pace range 40.6 ± 5.1% versus men 43.8 ± 7.5%, *p* = 0.037; pacing coefficient of variation *p* = 0.486. No significant MOMS-pacing correlations in either sex. Women’s CV prediction equation included goal achievement: +0.012 by goal achievement, *R* = 0.766, *p* < 0.001. Women scored higher than men on psychological coping, self-esteem, life meaning, health orientation, weight concern, and goal achievement.	Motivation dimensions were not clearly correlated with pacing, although goal achievement contributed to a women-only pacing prediction equation; women reported stronger motivation on several MOMS dimensions.
Philippe et al. (2016)	Qualitative comparison of common course-of-experience sequences across 10 ultra-trail runners who withdrew.	Seven representative sequences: feeling pain; putting meaning to feelings; adjusting running style; attempting to overcome the problem; other runners’ influences; assessing the situation; deciding to withdraw. Inter-coder agreement 70 to 90%.	Withdrawal was constructed as a progressive and cumulative process involving bodily, behavioral, cognitive, and social experiences.

**Table 7:** Findings of individual studies in the mental health, mood regulation, psychological resilience, and stress–response domain.

Study	Main comparison or exposure–outcome focus	Main statistical findings	Principal finding
Diotaiuti et al. (2021)	Structural equation model of autonomy satisfaction, competence satisfaction, locomotion mode, homeostatic reintegration, and resilient reintegration	Model fit χ^2^ = 872.152; CFI = 0.966; TLI = 0.952; RMSEA = 0.058. Competence→Autonomy SWE = 0.614, *p* < 0.001; Autonomy→Locomotion SWE = 0.574, *p* < 0.001; Competence→Homeostatic SWE = 0.489, *p* < 0.001; Locomotion→Resilient SWE = 0.379, *p* = 0.001; Homeostatic→Resilient SWE = 0.447, *p* < 0.001.	Basic psychological need satisfaction and self-regulatory locomotion were associated with resilience processes in endurance runners. The authors emphasize autonomy and competence as factors supporting perseverance, recovery, and psychophysical balance.
Gauld et al. (2024)	EAI and TIPI profile among ultrarunners hospitalized/dialysed after ultramarathon serious complications.	EAI text: M = 19.25, standard deviatio*n* = 4.95; 11 symptomatic (13–23; M = 18.8, standard deviatio*n* = 2.93, 1 at-risk (≥24), none asymptomatic. Table: S-EAI M = 19.30, standard deviatio*n* = 3.17. EAI centrality: mood modification most central across four metrics; withdrawal also highly central. TIPI emotional stability: M = 10.8 vs. 9.4; *t* = 2.2, *p* = 0.05.	Serious-complication ultrarunners were generally symptomatic but not mostly at-risk for exercise addiction. Mood modification and emotional stability were the most salient psychological features reported.
Graham et al. (2021)	Mental toughness and sleep quantity in relation to mood states and injury rate during a three-day 120-mile Arctic ultramarathon.	Injury rate differed by day: F(7,2) = 5.224, *p* = 0.041. Vigour changed over time: *F*(7,2) = 6.112, *p* = 0.029. Fatigue changed over time: F(7,2) = 8.303, *p* = 0.014. Mental toughness correlated with anger *r* = -0.61, confusion *r* = -0.55 to -0.56, depression *r* = -0.62, tension *r* = -0.42, and vigour *r* = 0.49/0.50; reported as significant. Mental toughness and injury rate: *r* = 0.14, not significant. Sleep and injury: *r* = -0.05, not significant; no significant sleep-mood relationships.	Mental toughness was associated with better mood regulation during the Arctic ultramarathon but did not predict injury rate. Sleep quantity was not related to mood or injury. Injuries were common and mood disruption increased during the race.
Howe et al. (2019)	Trait emotional intelligence and acute 80.5 km treadmill ultramarathon effects on total mood disturbance, serum cortisol, RPE, and completion time.	Whole-cohort TMD: *F*(1.4,11 = 7.8, *p* = 0.008, partial eta squared = 0.414. Cortisol: *F*(1.7,18.7) = 22.34, *p* < 0.001, partial eta squared = 0.67. Trait EI and post cortisol: *r* = 0.78, *p* < 0.01; high EI post cortisol 623.2 ± 134.5 vs. low EI 396.2 ± 109.1 ng/mL, *p* = 0.01. Low EI TMD increased baseline/pre to halfway, *p* = 0.02; post TMD high versus low EI *p* = 0.04. Trait EI and completion time: *p* > 0.05; run time group comparison *p* = 0.44. Controlled-velocity RPE group differences: 48.3 km *p* = 0.027 and 64.4 km *p* = 0.032.	Higher trait emotional intelligence was associated with better mood regulation and lower perceived exertion at controlled velocities, but with higher post-ultramarathon cortisol. Trait EI was not significantly associated with completion time.
Jaenes et al. (2022)	Associations between psychological wellbeing dimensions and competitive anxiety; moderated mediation model of marathon experience, wellbeing, age, and anxiety.	Somatic anxiety regression: self-acceptance beta = -0.249, *p* = 0.017; R2 = 0.097, *F*(9,237 = 2.729, *p* < 0.01. Worry regression: self-acceptance beta = -0.230, *p* = 0.024 and environmental mastery beta = -0.217, *p* = 0.012; R2 = 0.138, F(9,237) = 4.065, *p* < 0.001. Concentration disruption regression: environmental mastery beta = -0.290, *p* = 0.001; R2 = 0.109, F(9,237) = 3.107, *p* < 0.01. Mediation/moderation: wellbeing beta = -0.2970, 95% confidence interval [-0.4291, -0.1790]; indirect effect via wellbeing beta = -0.0530, 95% confidence interval [-0.0939, -0.0218]; marathons finished by age beta = 0.1560, 95% confidence interval [0.0696, 0.2385].	Higher psychological wellbeing was generally associated with lower competitive anxiety. Wellbeing partially mediated the relationship between greater marathon experience and lower anxiety, while age moderated the direct experience-anxiety relationship.
Krokosz et al. (2018)	Associations between personality traits or ultramarathon participation motives and mood states 12 h before a 100 km track ultramarathon.	Extraversion: energetic arousal rs = 0.47, *p* < 0.05; tense arousal rs = -0.48, *p* < 0.05. Neuroticism: tense arousal rs = 0.53, *p* < 0.05; hedonic tone rs = -0.57, *p* < 0.05. Company motive: energetic arousal rs = 0.63, *p* < 0.05; hedonic tone rs = 0.56, *p* < 0.05. Managing stress motive: tense arousal rs = 0.50, *p* < 0.05; escape motive: tense arousal rs = 0.46, *p* < 0.05.	Personality and selected motives were related to pre-race mood, but the authors reported that these relationships vanished 12 h after the race.
Lane and Wilson (2011)	High versus low trait emotional intelligence predicting mood states before and after each stage of a six-stage ultra-endurance foot race.	Trait emotional intelligence main effects: anger *F*(1,21) = 7.22, *p* = 0.001; calmness *F* = 6.05, *p* = 0.02; confusion *F* = 8.86, *p* = 0.01; depression *F* = 7.94, *p* = 0.01; fatigue *F* = 11.26, *p* = 0.00; happiness *F* = 14.22, *p* = 0.00; tension *F* = 6.11, *p* = 0.02; vigor *F* = 3.10, *p* = 0.09.	Higher trait emotional intelligence was associated with higher pleasant and lower unpleasant emotions across the multi-stage event; vigor did not reach statistical significance.
Larumbe-Zabala et al. (2020)	Within-runner longitudinal changes in Podium psychological variables and their associations during marathon preparation, with contextual physiological testing.	Perceived fitness to self-efficacy coefficient 1.14, 95% confidence interval 0.88 to 1.41, *p* < 0.001, f^2^ = 1.33. Self-efficacy to motivation coefficient 0.19, 95% confidence interval 0.11 to 0.27, *p* < 0.001, f^2^ = 0.36. Perceived fitness to motivation coefficient 0.21, 95% confidence interval 0.07 to 0.35, *p* = 0.003, f^2^ = 0.17. Self-efficacy to somatic anxiety coefficient -0.32, *p* = 0.008; perceived fitness to somatic anxiety coefficient -0.40, *p* = 0.03; perceived fitness to cognitive anxiety coefficient -0.81, *p* = 0.044; motivation to somatic anxiety coefficient -0.66, *p* = 0.048.	Perceived physical fitness, self-efficacy, and motivation were strongly interrelated over time, and increases in these positive psychological variables were generally associated with reductions in anxiety. Physiological variables improved over time, but relative changes in physiological parameters did not significantly predict perceived fitness changes.
Miller et al. (2023)	Cross-sectional regression and narrative accounts linking irrational performance beliefs, motivation regulation, and anxiety in elite ultramarathon runners.	Irrational beliefs predicted relative autonomy: b = -15.42, confidence interval -23.93 to -6.91, model R^2^ = 0.26. Irrational beliefs predicted composite anxiety: b = 0.66, confidence interval 0.33 to 1.00, model R^2^ = 0.24. Relative autonomy predicted composite anxiety after sex, age, and irrational beliefs: b = -0.26, confidence interval -0.38 to -0.15, model R^2^ = 0.44. Interview subgroup values: high irrational beliefs M = 18.75, standard deviatio*n* = 0.89; low irrational beliefs M = 9.67, standard deviatio*n* = 2.27.	Irrational performance beliefs and less autonomous motivation were associated with higher anxiety symptoms. Qualitative narratives suggested that irrational beliefs combined with controlled motivation may contribute to dysfunctional behaviors such as persisting through injury or withdrawing from sport.
Nicolas et al. (2019)	Trait emotional intelligence predicting immediate post-race sport emotions and one-month emotion trajectories after a mountain ultra-marathon.	Time effects: anxiety *β* = -0.12, *p* < 0.01; dejection *β* = -0.11, *p* < 0.01; anger *β* = 0.07, *p* < 0.05. Trait-EI general score predicted excitement *β* = 0.60, *p* < 0.05 and happiness *β* = 0.77, *p* < 0.05; Trait-EI by time predicted happiness *β* = -0.27, *p* < 0.05 and marginally excitement *β* = -0.16, *p* = 0.07.	Trait emotional intelligence was associated with higher positive emotion immediately after race completion and with distinct post-race emotional trajectories.
Nicolas et al. (2022)	High versus low trait emotional intelligence and recovery-stress states before, during, and after the Tor des Géants mountain ultra-marathon.	Recovery: time effect F(2,22 = 7.50, *p* = 0.003, partial η^2^ = 0.45; EI group by time *F*(2,22) = 12.21, *p* = 0.0003, partial η^2^ = 0.53; pre-race high EI versus low EI recovery *p* = 0.004, d = 4.35. Stress: EI group *F*(1,11) = 0.74, *p* = 0.408; EI by time *p* = 0.767; stress time effect F(2,22) = 5.19, *p* = 0.014.	High trait emotional intelligence was linked with higher pre-race recovery resources but did not significantly differentiate stress-state trajectories.
Popov et al., 2019	Multiple regression of six extracted motivation factors predicting positive affect, negative affect, and subjective well-being.	Models were significant: R2 = 0.22 for Positive Affect, R2 = 0.13 for Negative Affect, R2 = 0.23 for Subjective Well-Being. Mental Health Improvement: beta -0.31 for positive affect, beta 0.36 for negative affect, beta -0.36 for subjective well-being. Physical Health and Condition: beta 0.33 for positive affect, beta -0.22 for negative affect, beta 0.43 for subjective well-being.	Endurance running motivation was related to emotional well-being; coping with negative emotional states was a prominent motive and was associated with poorer concurrent well-being.
Tapia-Serrano et al., 2020)	Intrinsic and extrinsic motivation as mediators between expected race outcome and perceived health status.	Intrinsic motivation model: indirect beta = -0.013, standard erro*r* = 0.006, 95% confidence interval -0.026 to -0.002; expected outcome to intrinsic motivation beta = -0.093, *p* < 0.05; intrinsic motivation to perceived health beta = 0.142, *p* < 0.001. Extrinsic motivation model: indirect beta = -0.000, standard erro*r* = 0.001, 95% confidence interval -0.005 to 0.001.	Intrinsic motivation, but not extrinsic motivation, mediated the association between better expected race outcome and better perceived health status in long-distance runners.
Timm et al. (2017)	Qualitative resilience process after the 2013 Boston Marathon bombings.	Sixteen runners were interviewed 10 to 12 months after the event. Themes were: surreal race day; coping and processing emotional responses; and resulting impact. Fifteen of sixteen continued running, and thirteen planned to run the 2014 Boston Marathon.	Runners used mixed coping strategies and resources such as running, social support, previous experiences, perspective taking, and gratitude; positive outcomes included increased motivation, strength, new perspectives, and stronger running-community closeness.
Urquijo et al. (2024)	Emotion regulation and negative self-talk predicting perceived stress in trail runners.	Emotion regulation correlated with stress *r* = -0.403, *p* < 0.01 and with negative self-talk *r* = -0.208, *p* < 0.05; negative self-talk correlated with stress *r* = 0.433, *p* < 0.01. Mediation: direct emotion-regulation/stress B = -0.16, 95% confidence interval -0.23 to -0.09; indirect effect through negative self-talk = -0.03, 95% confidence interval -0.1536 to -0.0062.	Higher emotion regulation was associated with lower perceived stress, partly through lower negative self-talk. The study concluded that improving emotion regulation and redirecting negative self-talk may help manage trail runners’ stress.
van Iperen et al. (2022a)	Running-related demands-vigor associations moderated by running-related resources and recovery across physical, cognitive, and emotional dimensions.	Four significant matching moderations were reported across cognitive and emotional vigor. Cognitive liveliness: physical demands x physical recovery b = 0.20, *p* < 0.05; emotional demands x emotional recovery b = -0.23, *p* < 0.05. Emotional energy: emotional demands x emotional resources b = 0.17, *p* < 0.05; emotional demands x emotional recovery b = -0.25, *p* < 0.01. Matching-principle test H(2 = 0.002, *p* = 0.999.	Emotional resources and recovery were most relevant to vigor, but evidence was mixed and sometimes contrary to hypotheses; no evidence supported stronger effects for matched dimensions.
Woodman and Welch, (2022)	Alexithymia moderating pre- to post-race anxiety change in marathon and ultramarathon runners.	*N* = 35. Bootstrapped regression analyses using MEMORE revealed that alexithymia moderated the relationship between pre- and postrace anxiety; significant anxiety reduction was reported for individuals high in alexithymia only.	Extreme endurance running appeared to provide an anxiety-regulation function for runners high in alexithymia.

**Table 8:** Findings of individual studies in the performance outcomes and performance resilience domain.

Category	Study	Main comparison or exposure–outcome focus	Main statistical findings	Principal finding
Direct performance resilience indicator	Alschuler et al. (2020)	Pain coping composites predicting race completion and finishing-position quintile among the same 2016 RacingThePlanet cohort.	Logistic regression for odds of finishing: maladaptive pain coping odds ratio 0.32, 95% confidence interval 0.2-0.6, *p* < 0.001; adaptive pain coping odds ratio 0.72, 95% confidence interval 0.4-1.2, *p* = 0.2; experiential awareness odds ratio 1.25, 95% confidence interval 0.7-2.4, *p* = 0.5. Ordinal finishing-quintile analysis: no predictors related to higher or lower quintile, *p* > 0.2.	Lower use of maladaptive pain coping was associated with greater likelihood of race completion; coping composites were not associated with finishing-position quintile.
Direct performance resilience indicator	Brace et al. (2020)	Mental toughness and self-efficacy associations with each other and with Ultra-Trail World Tour rank, HURT100 completion, placing, and time.	Mental toughness and self-efficacy r(54 = 0.72, *p* < 0.001. Rank model *F*(2,53) = 0.738, *p* = 0.483. Completion model χ^2^ = 0.56, *p* = 0.756. Placing model F(2,53) = 1.738, *p* = 0.186. Time model *F*(2,30) = 2.046, *p* = 0.147. Sports Mental Toughness Questionnaire total mean 45.42 ± 4.26; higher than five comparison athlete groups, all *p* < 0.001.	Mental toughness and self-efficacy were strongly related but did not significantly predict elite ultramarathon rank, completion, placing, or finishing time in this sample, suggesting a possible threshold effect for elite participation rather than performance differentiation.
Direct performance resilience indicator	Corrion et al., 2018	Finishers versus non-finishers and multiple logistic regression of psychosocial predictors of dropout in 140 km ultra-trail runners.	MANOVA Wilks’ *λ* = 0.86, *F*(3,217) = 11.499, *p* < 0.001, η^2^ = 0.14. Self-efficacy: finishers M = 4.22, standard deviatio*n* = 0.69; non-finishers M = 3.87, standard deviatio*n* = 0.96; *F*(1,219) = 9.168, *p* < 0.01. Intention: finishers M = 4.34, standard deviatio*n* = 0.55; non-finishers M = 3.07, standard deviatio*n* = 0.88; F(1,219) = 20.685, *p* < 0.001. Avoidance coping: non-finishers M = 2.99, standard deviatio*n* = 0.89; finishers M = 2.59, standard deviatio*n* = 0.88; F(1,219) = 14.996, *p* < 0.001. Logistic regression explained 36.4%; ORs: started/finished ultra-trails 0.44, self-efficacy 2.03, intention 0.34, mastery-approach goals 0.56, seeking social support 0.43, avoidance coping 2.26.	Self-efficacy, intention to finish, mastery-approach goals, seeking social support, and experience were associated with lower dropout risk, whereas avoidance coping increased dropout risk; self-determined motivation and needs satisfaction were not associated with dropout.
Direct performance resilience indicator	de la Vega et al. (2011)	Hardy personality compared between mountain ultradistance and 10.06 km runners, and associated with ultradistance completion and race time.	Race-type comparison: total hardiness 71.88 ± 7.89 in mountain runners versus 71.37 ± 8.39 in 10.06 km runners, Z = -0.280, *p* = 0.779; commitment *p* = 0.890; control *p* = 0.593; challenge *p* = 0.735. Completion: finishers 71.93 ± 7.76 versus withdrawals 71.23 ± 8.37, Z = -0.278, *p* = 0.781. Race-time correlations among finishers: total *r* = -0.072, *p* = 0.309; components *p* = 0.352 to 0.386.	High levels of hardiness were observed in both endurance running groups, but hardiness did not distinguish ultra-trail finishers from withdrawals and was not related to faster ultra-trail times.
Direct performance resilience indicator	Méndez-Alonso et al., 2021	Ultra-trail runners’ mental toughness, resilience, harmonious passion, and obsessive passion in relation to race completion, race-time quartiles, ITRA score, age or experience, and pre- to post-race change.	Baseline means: mental toughness 6.84 (0.93), resilience 6.23 (0.65), harmonious passion 6.31 (0.83), obsessive passion 2.93 (1.16). Finishers versus withdrawals: mental toughness *t* = 4.25, *p* = 0.01; resilience *t* = 3.42, *p* = 0.01; harmonious passion *t* = -0.41, *p* = 0.01; obsessive passion *t* = -4.39 with printed *p* = 0.70. Race-time quartile ANOVA: mental toughness *F* = 18.121, *p* = 0.01; resilience *F* = 11.745, *p* = 0.01; harmonious passion *F* = 19.285, *p* = 0.01; obsessive passion *F* = 0.168, *p* = 0.682. pre- and post-race: mental toughness *t* = 2.35, *p* = 0.01; resilience *t* = 2.82, *p* = 0.01; harmonious passion *t* = 3.09, *p* = 0.01; obsessive passion *t* = -0.41, *p* = 0.99.	Mental toughness and resilience were associated with ultra-trail success, defined in terms of completion and race performance. Mental toughness, resilience, and harmonious passion increased after the race, suggesting that participation may reinforce these psychological resources.
Proximal indicator	Beattie et al., 2025	Moderation of the coping effectiveness-performance satisfaction relationship by mental toughness and resilience; coping frequency associations and trajectories; qualitative coping and satisfaction themes.	CE × mental toughness beta 1.37, ΔR2 = 0.09, *F*(1,44) = 4.47, *p* = 0.04, 95% confidence interval [0.06, 2.68]; adjusted *p* = 0.07. Coping effectiveness × CD-RISC beta 3.56, ΔR2 = 0.20, *F*(1,42) = 11.35, *p* = 0.001, 95% confidence interval [1.42, 5.69]; adjusted beta 3.85, *p* = 0.001. coping effectiveness × Brief Resilience Scale beta 2.00, ΔR2 = 0.11, *F*(1,43) = 5.47, *p* = 0.024, 95% confidence interval [0.27, 3.73]; adjusted beta 2.15, *p* = 0.021. Coping frequency-performance satisfaction *r* = -0.35, *p* < 0.05. Coping frequency time effect Wilks’ Lambda *F*(10,31) = 4.23, *p* < 0.001.	Coping effectiveness was positively related to performance satisfaction when mental toughness or resilience was high, but not when low. Coping frequency was higher among less satisfied participants and varied across race checkpoints.
General performance correlate	Christensen et al., 2018	Regression prediction of 161 km ultramarathon finishing time from demographic, training, psychological, and attentional variables.	*n* = 189 questionnaire respondents; *n* = 152 finishers; mean finish time 23:44:20; mean age 41.5 years; 126 men and 26 women among finishers. Slide results: sex and age did not significantly predict finish time; average weekly mileage was the best training predictor; Sports Mental Toughness Questionnaire, PCS, and SMS were not related to finish time; COCS attentional scores were significant, with more internal monitoring, outward monitoring, and outward distraction tending to faster finish times.	Evidence suggests attentional variables may relate to faster ultramarathon finishing time.
General performance correlate	Gameiro et al., 2023	Structural equation model of mental toughness, resilience, and International Trail Running Association Performance Index	Measurement model χ^2^ = 150.01(74), BS-*p* = 0.003, CFI = 0.953, TLI = 0.942, RMSEA = 0.058 90% confidence interval 0.045-0.071, SRM*R* = 0.042. Structural model χ^2^ = 150.01(75), CFI = 0.954, TLI = 0.944, RMSEA = 0.057 90% confidence interval 0.044-0.070, SRM*R* = 0.042. M*T* → resilience *β* = 0.77, 95% confidence interval 0.624-0.891, *p* = 0.002; resilience→performance *β* = 0.12, 95% confidence interval 0.010-0.210, *p* = 0.025; indirect M*T* → performance through resilience *β* = 0.09, 95% confidence interval 0.010-0.168, *p* = 0.02; R^2^ for performance = 21%.	Mental toughness was positively associated with resilience, resilience was positively associated with performance, and resilience was a possible mediator of the mental toughness-performance relationship.
General performance correlate	Gayton et al., 1986	Physical self-efficacy and subscales correlated with predicted and actual marathon finishing times.	Predicted time: total PSE *r* = -0.38, *p* < 0.05; perceived physical ability *r* = -0.47, *p* < 0.01; presentation confidence *r* = -0.17, *p* > 0.17. Actual time: total PSE *r* = -0.43, *p* < 0.01; perceived physical ability *r* = -0.55, *p* < 0.001; presentation confidence *r* = -0.17, *p* > 0.18.	Higher general physical self-efficacy, especially perceived physical ability, was associated with faster predicted and actual marathon finish times; presentation confidence was not associated.
General performance correlate	Gillet et al., 2012	Motivational profiles and motivation forms associated with final ranking and emotional/physical exhaustion in Marathon des Sables runners.	Final ranking by profile: Low 397.5 ± 207.2, Moderate 305.3 ± 234.4, High 260.7 ± 184.4; *F*(2,141) = 6.03, *p* < 0.01, η^2^ = 0.08; ANCOVA *p* < 0.05, η^2^ = 0.07. Exhaustion: Low 1.52 ± 0.53, Moderate 1.69 ± 0.60, High 2.09 ± 0.80; *F* = 10.93, *p* = 0.001, η^2^ = 0.13. Correlations with final ranking: identified *r* = -0.20, *p* < 0.05; external *r* = -0.24, *p* < 0.05; amotivation *r* = -0.18, *p* < 0.05; intrinsic and introjected not significant.	A High motivational profile was linked with better ranking but higher emotional/physical exhaustion, suggesting a performance–benefit/psychological cost pattern.
General performance correlate	Jaenes Sánchez et al., 2009	Higher versus lower hardiness among marathon runners; marathon runners versus a published university comparison sample.	Expected mark differed significantly by hardy-personality median split: G1 mean 207.25 min versus G2 mean 196.32 min, U = 2678.500, *p* = 0.021, d = -0.43. Obtained mark was not significant: *p* = 0.220. Total hardy personality was higher in marathoners than comparison sample: *p* = 0.024, d = 0.22; commitment *p* = 0.033, d = 0.21.	Marathon runners showed moderately high levels of hardiness. Greater hardiness was associated most clearly with more ambitious expected marathon performance, whereas differences in actual finishing time were not statistically significant.
General performance correlate	Kilduff, 2014	Race pace in seconds per kilometer when empirically identified rivals were present versus absent, plus count and continuous rivalry alternatives.	Rival present: beta -4.92 s per kilometer, standard error 1.39, t(1165) = -3.54, *p* < 0.001; number of rivals beta -1.89, standard error 0.55, *p* < 0.001; top rival present beta -2.83, standard error 1.33, *p* = 0.034; total rivalry beta -1.18, standard error 0.41, *p* = 0.004.	Runners had faster race pace in races featuring their rivals, supporting rivalry as a motivational exposure associated with improved performance.
General performance correlate	Lopez and Sánchez, 2023	Big Five personality profile of mountain/trail runners versus general-population norms and comparisons by distance, sex, motivation, and personality/performance clusters.	All runners versus general population: neuroticism -14.1, *p* < 0.01; extraversion +7.9, *p* < 0.05; openness +3.7, not significant; agreeableness -14.1, *p* < 0.01; conscientiousness +14.3, *p* < 0.01. Trail versus Ultra differences were not significant. Women versus men: only agreeableness differed significantly, women -11.8, *p* < 0.05. Cluster 2 had best race placement; Cluster 1 minus Cluster 2 place percent +32, *p* < 0.01.	Mountain runners showed low neuroticism and high conscientiousness, but not elevated openness. Personality differences by distance and sex were limited, and motivation or cluster analyses suggested possible but not definitive runner profiles.
General performance correlate	Pruszczak and Stolarski, 2025	Regression and mediation analyses testing sport time perspectives as predictors of official running performance.	Study 1: STPQ scales added 8% variance; Present-Fatalistic B = -20.08, *p* = 0.004; Future-Positive and Future-Negative also significant before anticipated performance was added. Study 2: Future-Positive B = 69.17, standard erro*r* = 21.55, beta = 0.33, *p* = 0.002. Mediation total indirect effect 59.61, 95% confidence interval 29.37 to 89.86; BMI, preparedness, and Vigor all significant mediators.	Future-Positive temporal framing consistently predicted better race performance; Present-Fatalistic predicted poorer 10-km performance; Vigor and preparedness helped explain the Future-Positive effect in the half-marathon sample.
General performance correlate	Rubaltelli et al., 2018	Structural equation model testing trait emotional intelligence, training load, prior experience, desired time, and expected time as predictors of official finish time.	Trait emotional intelligence to finish time: *β* = -0.69, *p* < 0.001. Trait emotional intelligence to desired time: *β* = -0.67, *p* < 0.001. Desired time to finish time: *β* = 0.12, *p* = 0.021. Expected time to finish time: *β* = 0.38, *p* < 0.001. Training load to finish time: *β* = -0.10, *p* = 0.006. Final model fit χ^2^(8) = 11.70, *p* = 0.17; R2 = 0.85.	Trait emotional intelligence was the strongest predictor of faster half-marathon finish time and was associated with more ambitious desired performance.
General performance correlate	Schüler and Brunner, 2009	Flow during marathon race or training predicting future running motivation, training behavior, and official marathon time across three field studies.	Study 1: race flow and future motivation *r* = 0.35, *p* < 0.01; race flow did not predict running time after intended time, beta = - 0.08, ns. Study 2: race flow predicted future motivation beta = 0.18, *p* < 0.05; race flow did not predict running time beta = - 0.08, ns; pre-race training behavior beta = - 0.23, *p* < 0.05. Study 3: training flow associated with training behavior *r* = 0.24, *p* < 0.05; direct training-flow/race-time beta = - 0.29, *p* < 0.05; adjusted beta = - 0.16, ns when training behavior was included.	Flow during the marathon was linked to future running motivation but not directly to race time. Flow during training appeared to support race performance indirectly via greater training behavior.
General performance correlate	Waleriańczyk, 2023	Perfectionistic strivings, perfectionistic concerns, their interaction, and 2 × 2 perfectionism profiles as predictors of transformed official trail-running performance.	Perfectionistic strivings B = 21.29, standard erro*r* = 4.79, beta = 0.38, 95% confidence interval 11.82 to 30.75, *p* < 0.001. Perfectionistic concerns direct effect B = -3.86, standard erro*r* = 4.10, beta = -0.08, 95% confidence interval -11.95 to 4.24, not significant. Strivings × concerns B = -3.61, standard erro*r* = 1.57, beta = -0.16, 95% confidence interval -6.71 to -0.50, *p* < 0.05. Model R2 = 0.27; interaction ΔR2 = 0.024. Predicted performance: pure personal standards 54.87, mixed 49.23, non-perfectionism 42.31, pure evaluative concerns 41.40.	Perfectionistic strivings were associated with better trail-running performance, but high perfectionistic concerns reduced or eliminated this benefit. Pure personal standards perfectionism was the most favorable profile.
General performance correlate	Waleriańczyk and Stolarski, 2021)	Perfectionistic strivings, perfectionistic concerns, Big Five traits, and anticipated performance predicting 10-km and half-marathon performance.	Study 1 10 km: strivings beta = 0.27, *p* < 0.001, ΔR2 = 0.070; strivings x anticipated performance beta = 0.07, *p* < 0.001. Study 2 half-marathon: strivings beta = 0.35, *p* < 0.001, ΔR2 = 0.136; with Big Five included beta = 0.35, *p* < 0.001; strivings x anticipated performance beta = 0.13, *p* = 0.015. Anticipated-performance models: strivings beta = 0.29 and 0.37 to 0.39, *p* < 0.001.	Perfectionistic strivings were positive predictors of distance-running performance and anticipated performance. Perfectionistic concerns were not direct predictors, and Big Five effects were limited.

**Table 9:** Findings of individual studies in the health, injury, and risk appraisal domain.

Study	Main comparison or exposure–outcome focus	Main statistical findings	Principal finding
Chalabaev et al., 2017	Associations of marathon-motivation indices with perceived susceptibility to injury and the perceived likelihood of continuing to run through pain.	Study 1: self-determination index *r* = -0.11, *p* = 0.035 with perceived injury susceptibility; controlled motivation index *r* = 0.13, *p* = 0.011; external regulation *r* = 0.11, *p* = 0.036; amotivation *r* = 0.12, *p* = 0.02. Study 2: self-determination predicted injury susceptibility *β* = -0.10, *p* = 0.049 and keep-running-through-pain susceptibility *β* = -0.11, *p* = 0.045; mediator predicted injury susceptibility *β* = 0.22, *p* < 0.001; external regulation predicted keep-running-through-pain susceptibility *β* = 0.11, *p* = 0.049.	Self-determined motivation was interpreted as protective, partly through a lower perceived likelihood of continuing to run through pain; controlled or external motivation was associated with a greater tendency toward risk-related continuation.
Masters and Ogles, 1998	Associations between association/dissociation cognitive strategies, motivation variables, injury, and performance across one retrospective and one prospective marathon-runner study.	Study 1: dissociation was not related to total previous-year injuries during training r (123) = -0.01, *p* > 0.05 or marathon r (121) = -0.07, *p* > 0.05. Study 2: association scores were higher in injured runners than non-injured runners, 50.28 versus 46.06, *F*(1,150) = 5.82, *p* < 0.05; cognitive index lower in injured runners, 93.59 versus 100.3, F(1,150) = 9.82, *p* < 0.01; cognitive index predicted injury after training variables, beta = 0.22, *t* = 2.76, *p* < 0.01. Cognitive index correlated with marathon time *r* = 0.28, *p* < 0.01 and with MOMS competition *r* = -0.32, *p* < 0.001.	Dissociation did not increase injury risk. Association/internal focus, especially among more competitive and invested runners, predicted later injury. Dissociation appeared linked with slower performance and less competitive/goal motivation.
van Iperen et al., 2022b	Low-, medium-, and high-risk psychological profiles compared for running-related injuries and chronic fatigue.	LPA selected a three-profile solution with entropy 0.810. Injury probabilities: low-risk 47%, medium-risk 59%, high-risk 71%; overall chi-square (2) = 7.753, *p* = 0.021; high-risk versus low-risk odds ratio = 2.684, confidence interval 1.286 to 5.603, *p* = 0.007. Chronic fatigue: overall chi-square (2) = 13.958, *p* = 0.001; low-risk was 0.60 standard deviation lower than medium-risk and 0.59 standard deviation lower than high-risk.	Patterns of passion, recovery, and resources distinguished psychological risk profiles. Low-risk runners had fewer running-related injuries and lower chronic fatigue than high-risk runners.

Table 6 summarizes studies in the cognitive, attentional, and self-regulatory processes domain.

Table 7 summarizes studies in the mental health, mood regulation, psychological resilience, and stress–response domain.

Findings related to performance were grouped according to their conceptual proximity to performance resilience. Race completion, non-completion, withdrawal, pacing maintenance under fatigue, and performance satisfaction in demanding race contexts were treated as direct or proximal performance resilience outcomes. Race time, rank, placing, and speed were treated as general performance correlates when the original study did not explicitly evaluate maintenance, deterioration, adaptation, or continuity under adversity. Table 8 summarizes studies in the performance outcomes and performance resilience domain.

Table 9 summarizes studies in the health, injury, and risk appraisal domain.

### 3.5 Certainty of the evidence

GRADE assessments for the principal quantitative exposure–outcome associations are presented in Table 10. Certainty was rated as low for the associations of maladaptive pain coping with pain interference and race completion, motivational quality with psychological wellbeing, anxiety, or perceived health, and perfectionistic characteristics with general running performance outcomes. Very-low-certainty evidence was identified for associations of emotional intelligence or emotion regulation with mood and stress outcomes, resilience-related constructs with performance and completion outcomes, and psychological characteristics with injury or risk-related continuation. The principal reasons for downgrading were methodological limitations in the contributing studies, inconsistency across populations and outcome measures, indirectness arising from the use of heterogeneous or generic performance outcomes, and imprecision associated with small or context-specific samples. No evidence body reached moderate or high certainty. Although the sensitivity synthesis excluding studies with serious methodological concerns preserved several of the principal directional patterns, certainty remained limited because most retained studies still had moderate methodological concerns, and several findings were supported by only one or a small number of studies. These ratings concern confidence in the presence and direction of the reported associations and should not be interpreted as evidence that modifying a psychological trait would cause improved running performance or psychological health.

**Table 10:** GRADE certainty of evidence for the principal quantitative associations between psychological characteristics and mental or performance resilience outcomes in long-distance and ultra-endurance runners.

Exposure–outcome association	Contributing studies	Summary of evidence	Risk of bias	Inconsistency	Indirectness	Imprecision	Publication bias	Certainty of evidence	GRADE interpretation
Maladaptive pain coping and pain interference or time spent thinking about pain	Alschuler et al. (2019); 1 prospective repeated-measures cohort	Higher maladaptive pain coping was associated with greater pain interference and more time spent thinking about pain at both the between-runner and within-runner levels. Experiential awareness was associated with less pain interference.	Serious^1^	Not serious^2^	Serious^3^	Not serious	Undetected	Low ⨁⨁◯◯	Maladaptive pain coping may be associated with poorer pain adaptation during multistage ultramarathon running.
Maladaptive pain coping and race completion	Alschuler et al. (2020); 1 prospective cohort	Greater maladaptive pain coping was associated with a lower likelihood of race completion, but coping measures were not associated with finishing-position quintile among finishers.	Serious^1^	Not serious^2^	Serious^3^	Not serious^4^	Undetected	Low ⨁⨁◯◯	Maladaptive pain coping may be associated with a lower likelihood of completing a multistage ultramarathon.
Emotional intelligence or emotion regulation and mood, perceived stress, or recovery-related outcomes	Lane and Wilson (2011); Howe et al. (2019); Nicolas et al. (2019, 2022); Urquijo et al. (2024); 5 studies	Higher emotional intelligence or emotion regulation was generally associated with more favorable mood, emotion, and perceived stress outcomes. Findings for cortisol, recovery resources, and stress trajectories were less consistent.	Serious^5^	Serious^6^	Not serious	Serious^7^	Undetected	Very low ⨁◯◯◯	The evidence is very uncertain regarding the association of emotional intelligence or emotion regulation with mood and stress responses.
Motivational quality or irrational performance beliefs and psychological well-being, anxiety, or perceived health	Popov et al. (2019); Tapia-Serrano et al. (2020); Miller et al. (2023); 3 studies	Autonomous or intrinsic motivation was associated with more favorable well-being or perceived health outcomes, whereas coping-oriented motives, controlled motivation, and irrational beliefs were associated with poorer concurrent well-being or greater anxiety.	Serious^8^	Not serious^9^	Serious^10^	Not serious	Undetected	Low ⨁⨁◯◯	Motivational quality may be associated with psychological well-being, anxiety, and perceived health, but directionality is uncertain.
Mental toughness, resilience, or self-efficacy and race completion, performance satisfaction, rank, or objective performance	Brace et al. (2020); Corrion et al. (2018); Gameiro et al. (2023); Méndez-Alonso et al. (2021); Beattie et al. (2025); de la Vega et al. (2011); 6 studies	Positive associations were reported for completion, performance satisfaction, and some performance indices, but null findings were observed for rank, placing, finishing time, and completion in elite or homogeneous samples.	Serious^11^	Serious^12^	Serious^13^	Not serious	Undetected	Very low ⨁◯◯◯	The evidence is very uncertain regarding whether mental toughness, resilience, or self-efficacy is associated with superior objective endurance running performance.
Perfectionistic strivings or concerns and general running performance	Waleriańczyk and Stolarski (2021); Waleriańczyk (2023); 2 analytical cross-sectional studies	Perfectionistic strivings were associated with better official running-performance indices, whereas perfectionistic concerns were not independently favorable and appeared to attenuate the association of strivings with performance.	Serious^14^	Not serious	Serious^15^	Not serious	Undetected^16^	Low ⨁⨁◯◯	Perfectionistic strivings may be associated with better general running-performance indices, but the evidence does not directly establish greater performance resilience.
Perfectionistic strivings or concerns and general running performance	Waleriańczyk and Stolarski (2021); Waleriańczyk (2023); 2 analytical cross-sectional studies	Perfectionistic strivings were associated with better official running-performance indices, whereas perfectionistic concerns were not independently favorable and appeared to attenuate the association of strivings with performance.	Serious^14^	Not serious	Serious^15^	Not serious	Undetected^16^	Low ⨁⨁◯◯	Perfectionistic strivings may be associated with better general running-performance indices, but the evidence does not directly establish greater performance resilience.
Psychological profiles, motivation, or attentional orientation and injury or risk-related continuation	Chalabaev et al. (2017); Masters and Ogles (1998); van Iperen et al. (2022a); 3 studies	Self-determined motivation was associated with lower perceived willingness to continue through pain; internal attentional focus was associated with subsequent injury in one cohort; and higher-risk psychological profiles were associated with more self-reported injuries and chronic fatigue.	Serious^17^	Serious^18^	Serious^19^	Not serious	Undetected	Very low ⨁◯◯◯	The evidence is very uncertain regarding associations between psychological characteristics and injury, unsafe continuation, or chronic-fatigue risk.

^1^ Downgraded one level because the evidence came from a single cohort with moderate methodological concerns, including potential limitations in exposure measurement, residual confounding, and event-specific sampling.

^2^ Consistency across independent studies could not be evaluated because only one cohort contributed to the evidence body. No additional downgrade was applied for inconsistency.

^3^ Downgraded one level because the evidence was derived from a single 250-km, six-stage desert ultramarathon cohort and may not transfer directly to road marathons, shorter trail races, elite runners, or other ultra-endurance formats.

^4^ Although only 28 non-completion events occurred, the confidence interval for the association remained entirely on the same side of the null threshold used for the association-focused GRADE assessment.

^5^ Downgraded one level because two contributing studies had serious methodological concerns and the remaining studies had moderate concerns. Several studies used small samples and subjective outcomes.

^6^ Downgraded one level because favorable associations with mood and perceived stress were not consistently accompanied by favorable cortisol, recovery, or stress-trajectory findings.

^7^ Downgraded one level because several contributing cohorts were small and provided limited information for distinguishing a null association from potentially meaningful associations across outcomes and assessment periods.

^8^ Downgraded one level because the evidence was predominantly cross-sectional or based on mediation models, with limited control of residual confounding and uncertain temporal ordering.

^9^ The direction of the findings was broadly coherent: more autonomous motivational regulation was generally associated with more favorable outcomes, whereas controlled motives or irrational beliefs were associated with less favorable outcomes.

^10^ Downgraded one level because motivational exposures and outcomes differed substantially across studies and included emotional well-being, anxiety symptoms, and self-reported perceived health rather than a common mental resilience endpoint.

^11^ Downgraded one level because one contributing study had serious methodological concerns and the remaining evidence had moderate concerns, including incomplete confounder control and heterogeneous exposure and outcome measurement.

^12^ Downgraded one level because studies reported both positive and null associations across completion, performance satisfaction, rank, race time, placing, and generic performance indices.

^13^ Downgraded one level because several outcomes were general performance correlates rather than direct indicators of performance maintenance or adaptation under adversity, and populations ranged from recreational trail runners to highly selected elite ultramarathon runners.

^14^ Downgraded one level because both studies had moderate methodological concerns and used observational analyses that could not establish temporal direction or adequately exclude residual confounding.

^15^ Downgraded one level because the reported outcomes were generic performance indices rather than direct measures of completion, pacing maintenance, adaptation, or performance continuity under adversity.

^16^ Both studies reported favorable associations and originated from a closely related research context, but this was not considered sufficient for an additional formal downgrade.

## 4 Discussion

This systematic review identified, appraised, and synthesized evidence on psychological traits and trait-like attributes associated with mental and performance resilience in long-distance and ultra-endurance runners. The evidence base was broad but unevenly developed. The most frequently studied constructs included motivation, coping, emotional intelligence or emotion regulation, mental toughness, psychological resilience, self-efficacy, personality dimensions, perfectionism, pain-related beliefs, and attentional or self-regulatory processes. Reported outcomes included pain adaptation, mood regulation, perceived stress, anxiety, cognitive function, race completion, race time, pacing, performance satisfaction, injury, and risk appraisal.

The principal contribution of this review is its integration of two strands of evidence that are often considered separately: psychological correlates of mental adaptation and psychological correlates of performance continuity under endurance stress. The synthesis indicates that psychological traits should not be interpreted as uniform predictors of superior running performance. Rather, they may influence how runners appraise exertion and pain, regulate affect, deploy coping strategies, make pacing and goal adjustment decisions, and persist or withdraw under adverse conditions. This distinction is important because the reported associations were often domain- and context-specific. For example, mental toughness, resilience, and self-efficacy were associated with performance or completion in some studies but were not consistently related to rank, finishing time, or outcomes in elite races (Brace et al., 2020; Corrion et al., 2018; Gameiro et al., 2023; Méndez-Alonso et al., 2021). Similarly, emotional intelligence and emotion regulation were more consistently associated with mood, perceived exertion, and stress-related outcomes than with objective performance endpoints (Howe et al., 2019; Lane and Wilson, 2011; Nicolas et al., 2019; Urquijo et al., 2024).

The evidence gap map further clarifies the field by showing that research is concentrated in a limited number of exposure–outcome pairs. Motivation, emotional intelligence or emotion regulation, psychological resilience, mental toughness, personality dimensions, coping, and perfectionism accounted for many mapped associations, whereas objective cognitive outcomes, psychophysiological stress responses, injury outcomes, risk-related persistence through pain, and processes leading to non-completion were comparatively understudied. The review therefore extends related syntheses of motivation, personality, mental health, and resilience in sport by focusing specifically on trait-level psychological attributes in long-distance and ultra-endurance running and by distinguishing mental resilience outcomes from performance resilience outcomes (Braschler et al., 2024; Gupta and McCarthy, 2022; Partyka and Waśkiewicz, 2024; Thuany et al., 2023; Waleriańczyk, 2023).

### 4.1 Pain adaptation and the psychological meaning of endurance discomfort

Evidence on pain suggests that resilience in endurance running involves more than just tolerating discomfort. In the repeated-measures RacingThePlanet cohort, maladaptive pain coping was consistently associated with a greater proportion of time spent thinking about pain and with greater pain interference during multistage ultramarathon stages, whereas experiential awareness was associated with less pain interference (Alschuler et al., 2019). In a related performance analysis of the same race series, lower maladaptive pain coping was associated with a greater likelihood of race completion, although coping composites were not associated with finishing-position quintile among finishers (Alschuler et al., 2020). These findings suggest that coping quality may be more relevant to maintaining engagement and reducing interference than to differentiating rank among athletes who complete the event.

Experimental and comparative studies have reported greater cold-pain tolerance and lower pain-related anxiety among ultramarathon runners than among comparison participants (Freund et al., 2013; Roebuck et al., 2018). However, both studies were classified as having serious methodological concerns and were therefore removed from the risk-of-bias sensitivity synthesis. Their findings should consequently be regarded as preliminary and should not be interpreted as stable evidence that participation in ultramarathon running produces greater pain tolerance or that pain-related psychological characteristics causally explain endurance performance. Freund et al. (2013) also found no association between general self-efficacy and cold-pain tolerance, suggesting that broad confidence-related constructs may not correspond directly to experimentally assessed pain responses. Overall, the more defensible signal in this domain concerns the association of maladaptive pain coping with pain interference and completion rather than a generalized pain-tolerance advantage among ultrarunners.

Qualitative studies provide an important interpretive counterpoint by showing that pain resilience is not simply a psychometric trait or sensory threshold. Bennett et al. (2026) described women ultrarunners’ pain experiences as relational, embodied, and dynamically reinterpreted during a six-day ultramarathon, with pain shifting from a problem requiring resolution to a chosen experience supported collectively. Gross (2025) similarly interpreted ultrarunners’ accounts of pain and suffering through moral language that linked suffering to self-discipline, perseverance, identity, and meaningful achievement. These findings help explain why the same physical sensations may be experienced as threatening, manageable, meaningful, or identity-confirming, depending on the context. They also caution against treating pain tolerance as inherently adaptive. In endurance settings, psychological strategies that support persistence may benefit performance continuity, but they may also encourage unsafe persistence if pain is normalized without adequate attention to injury signals or medical risk.

### 4.2 Cognitive control, attentional strategies, and self-regulatory flexibility

Evidence on cognitive, attentional, and self-regulatory processes indicates that endurance performance is partly shaped by how runners monitor internal cues, interpret discrepancies between goals and current performance, and adjust behavior under fatigue. Brick et al. (2015) identified metacognitive planning, monitoring, reviewing, feelings, and judgments as central processes through which endurance runners regulate discomfort and performance. Buman et al. (2008) showed that “hitting the wall” is a multidimensional experience and that runners use cognitive, physical or behavioral, and emotion-focused strategies, as well as willpower, although many reported limited or ineffective coping responses. Together, these studies suggest that cognitive resilience in running depends less on a single attentional style than on the ability to select and revise strategies flexibly as demands change.

The evidence also suggests that persistence and disengagement should not be treated as simple opposites. Jackman et al. (2024) found that runners who reported excellent performances used mental contrasting with implementation intentions to decide whether to persist with a goal, revise it, or disengage and re-engage with an alternative. By contrast, Philippe et al. (2016) characterized withdrawal as a progressive, cumulative process involving bodily, behavioral, cognitive, and social experiences. These findings imply that adaptive performance resilience may include goal revision and strategic disengagement when initial goals become unrealistic or unsafe. This consideration is particularly important in ultra-endurance contexts, where rigid persistence may sustain effort but also increase exposure to injury, medical complications, or psychological distress.

Quantitative evidence on attention and pacing is consistent with this interpretation. Deaner et al. (2019) found that pace-related risk-taking was the most robust psychological predictor of slowing during the second half of a marathon after adjustment for covariates; willingness to suffer, competitiveness, goal achievement, and broader risk-taking indices were not robust adjusted predictors. Masters and Lambert (1989) reported that associative strategies and drive or competition were associated with faster marathon performance, whereas dissociation was not linked to injury indicators. However, Masters and Ogles (1998) found that association or internal focus, particularly among more competitive and invested runners, predicted subsequent injury. This pattern underscores the ambivalent role of attentional focus: monitoring bodily cues may facilitate pacing and effort regulation, but sustained internal focus among highly driven runners may also be associated with injury risk. The most defensible interpretation is therefore not that association or dissociation is universally superior but that attentional strategies interact with motivation, goal demands, pain appraisal, and situational risk.

Objective evidence on cognitive outcomes remains limited. Boere et al. (2026) reported changes in behavioral and electroencephalographic indices of executive function after a 50-km ultramarathon, with pre-race motivation and psychological distress associated with larger neural reductions. Although race-induced changes do not establish causal associations between traits and outcomes, this study extends the literature beyond self-report by linking psychological attributes with neurocognitive adaptation after acute endurance load. Further research is needed to determine whether cognitive resilience under fatigue is predicted by stable traits, transient psychological states, training history, or interactions among these factors.

### 4.3 Mental resilience, emotional regulation, and psychological health

Evidence on mental resilience focused primarily on mood regulation, perceived stress, psychological wellbeing, anxiety, recovery resources, and mental health symptoms. Emotional intelligence and emotion regulation formed one of the clearest thematic clusters. Lane and Wilson (2011) reported that higher trait emotional intelligence was associated with more pleasant and fewer unpleasant emotions during a multistage event. Howe et al. (2019) found that trait emotional intelligence was associated with better mood regulation and lower perceived exertion at controlled velocities; however, it was also associated with higher post-ultramarathon cortisol, complicating a uniformly protective interpretation. Nicolas et al. (2019, 2022) suggested that emotional intelligence may be associated with positive affect and recovery resources but not with all trajectories of stress states. Urquijo et al. (2024) further found that emotion regulation was associated with lower perceived stress, partly through lower negative self-talk. The sensitivity synthesis retained the general association of emotional intelligence or emotion regulation with affective and perceived stress outcomes after studies with serious methodological concerns were excluded, although evidence concerning cortisol and recovery-related responses became narrower. The evidence supports emotional regulation as a plausible domain of relevance but does not establish that higher emotional intelligence produces better physiological recovery, mental resilience, or running performance.

Motivation and psychological wellbeing also emerged as important, although their interpretation depends on motivational quality and outcome context. Popov et al. (2019) found that motivation for endurance running was associated with emotional wellbeing; coping with negative emotional states was a prominent motive associated with poorer concurrent wellbeing. Tapia-Serrano et al. (2020) reported that intrinsic motivation mediated the association between a more favorable expected race outcome and perceived health status, whereas extrinsic motivation did not. Miller et al. (2023) found that irrational performance beliefs and less autonomous motivation were associated with greater anxiety symptoms in elite ultramarathon runners; qualitative accounts also suggested links with maladaptive behaviors, such as persisting through injury or withdrawing from sport. These findings are consistent with the interpretation that autonomous, meaning-based, or intrinsically regulated motives may be more compatible with mental resilience than externally controlled or anxiety-driven motives. Nevertheless, because most evidence came from cross-sectional mediation and profile analyses, the direction of causation remains uncertain.

Studies of resilience, mental toughness, and need satisfaction further emphasize the relational and contextual nature of mental resilience. Diotaiuti et al. (2021) linked autonomy, competence, self-regulatory locomotion, and resilience processes in endurance runners, suggesting that the satisfaction of psychological needs may support adaptive reintegration after stress. Graham et al. (2021) found that mental toughness was associated with better mood regulation during a multiday Arctic ultramarathon but not with injury rates. Beattie et al. (2025) found that coping effectiveness was more strongly associated with performance satisfaction when mental toughness or resilience was high, suggesting that trait-like resources may shape how coping translates into satisfaction. These findings are consistent with the interpretation that autonomous, meaning-based, or intrinsically regulated motives may be more compatible with mental resilience than externally controlled or anxiety-driven motives. Temporal ordering, therefore, remains uncertain since motivational regulation may influence wellbeing and anxiety, but previous experiences of performance, distress, injury, or participation in endurance running may also shape athletes’ reported motives and beliefs.

Evidence on mental health also indicates that endurance running may be associated with vulnerability as well as adaptation. Gauld et al. (2024) reported symptoms of exercise addiction among ultrarunners who were hospitalized or dialyzed after serious ultramarathon complications, although the small, selected rare-event sample limits generalizability. Woodman and Welch (2022) suggested that extreme endurance running may serve an anxiety-regulation function for runners with high alexithymia. These findings should not be interpreted as evidence that ultrarunning is inherently harmful or therapeutic. Rather, they indicate that psychological traits may influence why individuals engage in extreme endurance running and how that engagement relates to mental health. This dual role is important for practitioners because the same behavior that supports identity, emotion regulation, and community connection may become maladaptive when driven by uncontrolled motives, rigid beliefs, or a limited ability to disengage.

### 4.4 Performance resilience, completion, and the limits of trait-based prediction

Evidence on performance resilience indicates that psychological traits and coping processes are associated with race completion, race time, ranking, pacing, and performance satisfaction, but not uniformly or deterministically. In ultra-trail and multistage ultramarathon contexts, lower maladaptive coping, greater self-efficacy, a stronger intention to finish, mastery-approach goals, social support seeking, mental toughness, resilience, and harmonious passion were associated with completion or performance-related outcomes in some studies (Alschuler et al., 2020; Corrion et al., 2018; Méndez-Alonso et al., 2021). Gameiro et al. (2023) reported that mental toughness was positively associated with resilience and that resilience was associated with performance, suggesting a possible pathway linking psychological resources with performance indices. Beattie et al. (2025) further reported that mental toughness and resilience moderated the association between coping effectiveness and performance satisfaction. These findings suggest that psychological resources may be most relevant when performance is conceptualized as persistence, effective coping, or satisfaction under demanding conditions.

Interpretation of this evidence depends on the nature of the performance endpoint. Race completion, non-completion, withdrawal, and maintenance of pacing under fatigue directly or proximally represent persistence and continuity during endurance stress. By contrast, race time, rank, placing, and speed are general performance outcomes unless the study explicitly demonstrates maintenance, disruption, or adaptation under adversity. These outcomes are strongly influenced by physiological fitness, training history, biomechanics, nutrition, race strategy, environmental conditions, and the competitive field. Consequently, an association between a psychological trait and faster performance was not considered sufficient, in isolation, to demonstrate greater performance resilience.

An important interpretive difference concerns the nature of the performance endpoint. Completion, withdrawal, pacing maintenance, and performance satisfaction under demanding conditions more directly reflect persistence or adaptation during endurance stress. By contrast, race time, rank, placing, and speed may primarily reflect general performance capacity and are influenced by physiological fitness, training history, biomechanics, nutrition, race strategy, competition level, and environmental conditions. Accordingly, an association between a psychological trait and a faster finishing time was not considered sufficient, in isolation, to demonstrate greater performance resilience.

By contrast, evidence from elite and highly selected ultramarathon samples illustrates the limits of trait-based prediction. Brace et al. (2020) found that mental toughness and self-efficacy were strongly correlated but did not significantly predict Ultra-Trail World Tour rank, HURT100 completion, placing, or finishing time. This null pattern may reflect restricted variability among elite runners, the overriding influence of physiological capacity and race-specific experience, or the possibility that mental toughness and self-efficacy are threshold characteristics for participation rather than variables that discriminate performance within elite samples. Similarly, de la Vega et al. (2011) found high levels of hardiness in endurance running groups, but hardiness did not differentiate ultra-trail finishers from those who withdrew or distinguish faster from slower finishers. These findings argue against a simplistic model in which higher scores on resilience-related traits automatically translate into better performance.

Studies of race time and ranking also highlight the diverse pathways through which psychological traits may relate to performance. Gayton et al. (1986) found that general physical self-efficacy, particularly perceived physical ability, was associated with predicted and actual marathon finishing times. Rubaltelli et al. (2018) reported that trait emotional intelligence predicted faster half-marathon finishing times and more ambitious performance goals. Waleriańczyk (2023) and Waleriańczyk and Stolarski (2021) found that perfectionistic strivings were associated with better distance- or trail-running performance, whereas perfectionistic concerns weakened or eliminated this benefit. Gillet et al. (2012) identified a high-motivation profile associated with better ranking but also with greater emotional and physical exhaustion, suggesting a potential performance benefit accompanied by a psychological cost. Kilduff (2014) showed that rivalry was associated with a faster race pace, whereas Schüler and Brunner (2009) linked flow during training and racing with motivational and performance-related pathways. Thus, confidence, ambition, motivational intensity, rivalry, perfectionistic striving, and flow may influence performance resilience through training behavior, goal setting, pacing, and competitive arousal while carrying different implications for mental health.

The sensitivity synthesis retained the overall conclusion that mental toughness, resilience, and self-efficacy show heterogeneous and context-dependent associations with running outcomes. Positive associations remained for selected completion, satisfaction, or general performance outcomes, whereas null findings persisted for race time, placing, rank, and completion in elite or homogeneous samples. GRADE certainty for this body of evidence was very low because of methodological limitations, inconsistent findings, heterogeneous construct measurement, and indirectness arising from the inclusion of generic performance outcomes. Evidence concerning perfectionistic strivings and general performance was graded as low certainty and did not directly establish performance resilience under adversity. Psychological characteristics may therefore be relevant to coping, goal regulation, pacing decisions, and performance satisfaction, but the current evidence does not support their use as stand-alone predictors of objective endurance performance or demonstrate that increasing these traits would improve race outcomes.

### 4.5 Health, injury, and risk appraisal as underdeveloped but clinically important domains

Health, injury, and risk appraisal were among the least populated domains in the evidence map, yet they are central to a responsible interpretation of resilience. Chalabaev et al. (2017) found that self-determined motivation was associated with a lower perceived likelihood of continuing to run through pain, whereas controlled or external motivation was associated with a greater tendency toward risk-related continuation. Masters and Ogles (1998) reported that dissociation did not increase injury risk, but association or internal focus, particularly among more competitive and invested runners, predicted subsequent injury. Van Iperen et al. (2022a) identified psychological profiles characterized by passion, recovery, and resources; runners in the low-risk profile had fewer running-related injuries and less chronic fatigue than those in the high-risk profile. By contrast, Graham et al. (2021) found that mental toughness was associated with mood regulation but did not predict injury rates during an Arctic ultramarathon.

The available evidence raises the possibility that psychological characteristics supporting commitment and persistence may also contribute to unsafe continuation when athletes ignore warning signs, interpret withdrawal as failure, or normalize clinically relevant pain. Conversely, flexible coping and recovery-oriented profiles may assist runners in distinguishing tolerable discomfort from symptoms requiring adjustment or withdrawal. However, GRADE certainty for psychological characteristics in relation to injury or risk-related continuation was very low. The evidence was sparse, exposures and outcomes were not directly comparable, and several findings relied on self-reported injury, hypothetical continuation decisions, or studies with serious methodological concerns. The association between motivational regulation and willingness to continue through pain, for example, was no longer represented after the exclusion of studies with serious concerns. This domain should therefore be viewed as clinically important but hypothesis-generating. Current evidence does not support using psychological profiles to predict injury or determine whether an athlete will persist unsafely.

### 4.6 Interpretation in light of methodological quality and evidence mapping

The methodological appraisal substantially constrains confidence in the synthesized evidence. Of the 53 included studies, 36 had moderate methodological concerns, and 14 had serious concerns; only 3 were classified as having minor concerns. The minor-concern category should not be interpreted as a complete absence of potential bias. Rather, it indicates that the identified limitations were considered unlikely to materially alter the principal interpretation. Common concerns included convenience or highly selected sampling, limited control of confounding, uncertain temporal ordering, subjective or incompletely validated exposure and outcome measures, small samples, and incomplete reporting of follow-up or attrition. Qualitative studies generally demonstrated congruity among their research questions, methods, analyses, and conclusions, but researcher reflexivity, researcher influence, and ethical reporting were often incompletely described.

The risk-of-bias sensitivity synthesis clarified which findings remained after excluding the 14 studies with serious methodological concerns. The principal patterns involving maladaptive pain coping, emotional regulation and affective or perceived stress outcomes, and the heterogeneous relationship between resilience-related constructs and performance remained evident in the restricted synthesis. Their persistence indicates that these conclusions were not solely produced by the studies with the most consequential methodological limitations. However, 36 of the 39 retained studies still had moderate concerns, and only 3 had minor concerns. Stability after exclusion should, therefore, be interpreted as relative robustness rather than confirmation that the remaining evidence is free from bias.

Several secondary findings were not robust to this restriction. Comparative evidence concerning greater cold-pain tolerance and lower pain-related anxiety among ultramarathon runners was removed because the contributing studies had serious methodological concerns. The same applied to selected findings concerning motivational regulation and continuation through pain, exercise addiction symptoms in runners with serious medical complications, alexithymia-related anxiety regulation, and several isolated performance associations. These observations remain relevant for hypothesis generation, but they should not be presented as established characteristics of long-distance or ultra-endurance runners.

The GRADE assessment provided a separate evaluation of certainty across each principal body of quantitative evidence. Certainty was low for the associations of maladaptive pain coping with pain interference and completion, motivational quality with psychological wellbeing or anxiety, and perfectionistic characteristics with general performance. Certainty was very low for emotional intelligence or emotion regulation in relation to mood and stress responses, resilience-related constructs in relation to performance or completion, and psychological characteristics in relation to injury or risk-related continuation. No evidence body reached moderate or high certainty. The principal reasons for downgrading were methodological limitations, inconsistency, indirectness, and imprecision.

These ratings concern confidence in the existence and direction of an association, not confidence in causality. Most included studies were observational, and many were cross-sectional or retrospective. Temporal precedence was consequently unresolved, residual confounding remained plausible, and reverse causation could not be excluded. For example, an association between mental toughness and performance does not determine whether mental toughness influences subsequent performance, whether successful participation and previous performance alter self-reported mental toughness, or whether both reflect common determinants such as training history, competitive level, prior success, and self-selection into endurance running. The available evidence, therefore, does not establish that increasing mental toughness, resilience, emotional intelligence, or another psychological characteristic through training will improve endurance running performance or mental health.

The evidence map should be interpreted in conjunction with these certainty judgments. Motivation was the most frequently represented exposure category, but it encompassed conceptually different constructs, including autonomous and controlled motivation, achievement goals, rivalry, passion, and motives related to coping with negative emotions. Emotional intelligence and emotion regulation were concentrated around mood and stress outcomes, whereas objective performance, injury, and safe withdrawal outcomes were less frequently examined. Mental toughness and resilience were prominent constructs, but their associations with objective performance remained inconsistent.

### 4.7 Practical implications for sport psychology, coaching, and endurance running support

The findings have practical implications but should be applied cautiously. For sport psychologists, coaches, and endurance support teams, flexible coping, emotion regulation, self-talk, goal adjustment, autonomous motivation, and pain appraisal should be regarded as candidate domains for individualized assessment and future intervention testing, rather than as established performance-enhancing targets. The available associations may inform hypothesis generation and case formulation, but they do not demonstrate that changing these characteristics will improve race completion, pacing, finishing time, performance satisfaction, or mental health. Developing and testing approaches that help runners distinguish manageable discomfort from pain signaling clinically relevant risk may be particularly important in ultra-endurance contexts. Performance satisfaction and persistence may reflect not only levels of traits such as mental toughness or resilience but also the ability to deploy effective coping strategies under race-specific demands (Beattie et al., 2025; Corrion et al., 2018).

From an applied perspective, psychological assessment should guide individualized support rather than label athletes as resilient or non-resilient. Mental toughness, resilience, emotional intelligence, perfectionism, and self-efficacy may provide useful information when interpreted alongside training history, injury status, sleep, nutrition, race experience, environmental exposure, and current mental health. The review also cautions against portraying endurance-related suffering as inherently virtuous. Qualitative evidence indicates that meaning-making around suffering can support identity and persistence, whereas evidence on risk appraisal suggests that externally controlled motives, rigid beliefs, and difficulty disengaging may contribute to harmful persistence (Chalabaev et al., 2017; Gross, 2025; Miller et al., 2023). Where these skills are addressed in practice, psychological support should balance adaptive persistence with appropriate disengagement; however, their effects on performance, mental health, and athlete safety require direct evaluation in intervention studies.

The findings also support greater standardization of psychological constructs and outcome definitions in measurement and evidence synthesis. Performance resilience should not automatically be equated with a faster race time. Completion, maintenance of pacing, performance satisfaction, adaptive goal revision, recovery, and safe withdrawal may represent distinct but equally important forms of endurance adaptation. Similarly, mental resilience should be conceptualized in terms of distinct dimensions, including mood regulation, stress tolerance, wellbeing, anxiety, coping effectiveness, pain adaptation, and recovery resources. A more differentiated measurement approach would help practitioners and researchers avoid conflating desirable performance outcomes with psychologically healthy adaptation.

### 4.8 Limitations of the included evidence base and of the review process

The limitations of the evidence base constrain interpretation. Most studies were observational, and many were cross-sectional or retrospective; consequently, the direction of associations between psychological traits and outcomes is often uncertain. The available designs cannot distinguish whether psychological traits precede and influence running outcomes, whether running experience and performance or injury histories alter psychological self-perceptions, or whether both are explained by common determinants. They also do not permit the inference that modifying a psychological trait through training would improve performance. Several studies used self-selected or small samples, event-specific cohorts, male-dominated samples, or highly selected groups of ultramarathon runners. These sampling features limit generalizability across sex and gender groups, competitive levels, race distances, terrain types, cultural contexts, and recreational versus elite populations.

Measurement heterogeneity was another major limitation. Studies differed in how they defined and measured mental toughness, resilience, motivation, emotional intelligence, coping, personality, self-efficacy, and perfectionism. Some studies reported only race time, rank, placing, or speed without assessing whether performance was maintained or adapted under fatigue, pain, environmental exposure, or another identifiable stressor. These outcomes were therefore retained as general performance correlates but were not considered direct evidence of performance resilience. Although this distinction reduced construct overinterpretation, it also limited the amount of evidence that could be classified as directly addressing performance resilience. Together with heterogeneity in psychological constructs, outcome definitions, and analytical methods, this precluded a meta-analysis and necessitated a conservative narrative synthesis.

### 4.9 Research gaps and priorities for future studies

The most important research priority is to move from broad correlational evidence toward prospective, preregistered, and mechanistically informed studies. Future research should assess psychological traits before events, repeatedly capture state-level processes during events, and link these data with objectively measured completion, pacing, race time, injury, medical events, recovery, and mental health outcomes. Multilevel designs are particularly appropriate for ultramarathons and multistage races because they can distinguish between-runner differences from within-runner changes in pain, coping, affect, fatigue, and goal adjustment.

Future studies should also improve control of confounding variables. Psychological constructs should be analyzed alongside training volume, race experience, history of non-completion, injury history, baseline fitness, sleep, nutrition, environmental exposure, sex or gender, age, competitive level, and race difficulty. Performance studies should distinguish among predicting faster times, maintaining pacing, completing events, achieving personally meaningful goals, and withdrawing safely. Studies of mental resilience should distinguish among short-term mood regulation, recovery resources, anxiety, perceived stress, wellbeing, and clinically relevant symptoms. Without these distinctions, the field will continue to conflate different forms of adaptation.

Intervention research is also needed. The included evidence suggests plausible targets, including adaptive pain coping, emotion regulation, negative self-talk, goal revision, autonomous motivation, coping effectiveness, and risk appraisal. However, the current evidence does not establish which psychological skills-training approaches improve mental resilience, performance resilience, or safety in long-distance and ultra-endurance runners. Randomized trials and pragmatic field studies should test whether targeted psychological interventions improve coping, satisfaction, safe completion, recovery, or mental health without promoting harmful persistence despite injury or medical warning signs.

Greater population diversity is another priority. Women and gender-diverse runners, younger and older runners, athletes with disabilities, runners from non-Western contexts, and athletes across competitive levels remain insufficiently represented in many evidence clusters. Qualitative and mixed-methods studies can help clarify how identity, gender, culture, social support, race organization, and community norms shape the meaning of endurance-related suffering and resilience.

Future studies should define performance resilience prospectively and distinguish it from general running performance. Direct outcomes should include completion or non-completion, pacing maintenance under fatigue, adaptation following disruption, safe withdrawal, and recovery after adversity, rather than relying solely on race time, rank, placing, or speed. Prospective and multilevel designs should establish temporal ordering by assessing psychological characteristics before events and repeatedly measuring coping, affect, pain, pacing, and decision-making during competition. Randomized and pragmatic intervention studies are required before claims can be made that modification of a psychological characteristic improves performance, mental health, or safety.

Future systematic reviews should preserve all pre-consensus reviewer decisions, report stage-specific inter-reviewer agreement, use external adjudication for unresolved or conceptually ambiguous cases where feasible, and prespecify certainty and sensitivity procedures in the review protocol. Greater standardization of psychological constructs and outcome definitions would also permit more informative quantitative synthesis and stronger certainty assessments.

## 5 Conclusion

This systematic review indicates that psychological traits and trait-like attributes are associated with several dimensions of mental and performance resilience in long-distance and ultra-endurance runners, but the evidence is heterogeneous and predominantly observational. The clearest patterns indicate that maladaptive pain coping is associated with greater pain interference and a lower likelihood of completion; emotional intelligence and emotion regulation are associated with mood- and stress-related outcomes; motivational quality is relevant to wellbeing and risk appraisal; and resilience-related constructs may support coping effectiveness and performance satisfaction. Evidence that these traits directly predict objective performance, particularly among elite or highly selected runners, is less consistent. Methodological concerns were common, especially in relation to exposure measurement, confounding, attrition, and subjective outcomes. The findings therefore support a cautious, domain-specific interpretation: psychological traits are associated with how runners report and regulate pain, emotion, cognition, motivation, and persistence under endurance stress, but the current evidence neither establishes causal effects nor demonstrates that modifying these traits improves endurance running performance. Future research should prioritize prospective, standardized, diverse, and intervention-oriented designs that distinguish adaptive persistence from unsafe continuation.

## Data Availability

The original contributions presented in the study are included in the article/supplementary material, further inquiries can be directed to the corresponding author/s.
